# Immature myeloid progenitors promote disease progression in a mouse model of Barrett's-like metaplasia

**DOI:** 10.18632/oncotarget.5431

**Published:** 2015-10-12

**Authors:** Jianping Kong, Hong Sai, Mary Ann S. Crissey, Nirag Jhala, Gary W. Falk, Gregory G. Ginsberg, Julian A. Abrams, Hiroshi Nakagawa, Kenneth Wang, Anil K. Rustgi, Timothy C. Wang, John P. Lynch

**Affiliations:** ^1^ Division of Gastroenterology, Department of Medicine, University of Pennsylvania, Philadelphia, PA, USA; ^2^ Department of Pathology, Temple University, Philadelphia, PA, USA; ^3^ Division of Gastroenterology, Columbia University, New York, NY, USA; ^4^ Division of Gastroenterology, Mayo Clinic, Rochester, MN, USA

**Keywords:** Barrett's esophagus, myeloid-derived suppressor cells (MDSC), IL-17, S100A9, IL-1β

## Abstract

Cdx2, an intestine specific transcription factor, is expressed in Barrett's esophagus (BE). We sought to determine if esophageal Cdx2 expression would accelerate the onset of metaplasia in the *L2-IL-1β* transgenic mouse model for Barrett's-like metaplasia. The *K14-Cdx2::L2-IL-1β* double transgenic mice had half as many metaplastic nodules as control *L2-IL-1β* mice. This effect was not due to a reduction in esophageal IL-1*β* mRNA levels nor diminished systemic inflammation. The diminished metaplasia was due to an increase in apoptosis in the *K14-Cdx2::L2-IL-1β* mice. Fluorescence activated cell sorting of immune cells infiltrating the metaplasia identified a population of CD11b^+^Gr-1^+^ cells that are significantly reduced in *K14-Cdx2::L2-IL-1β* mice. These cells have features of immature granulocytes and have immune-suppressing capacity. We demonstrate that the apoptosis in *K14-Cdx2::L2-IL-1β* mice is CD8^+^ T cell dependent, which CD11b^+^Gr-1^+^ cells are known to inhibit. Lastly, we show that key regulators of CD11b^+^Gr-1^+^ cell development, IL-17 and S100A9, are significantly diminished in the esophagus of *K14-Cdx2::L2-IL-1β* double transgenic mice. We conclude that metaplasia development in this mouse model for Barrett's-like metaplasia requires suppression of CD8+ cell dependent apoptosis, likely mediated by immune-suppressing CD11b^+^Gr-1^+^ immature myeloid cells.

## INTRODUCTION

Esophageal adenocarcinoma (EAC) is an important cause of death in the US, and one of a few cancers whose incidence is on the rise [1]. EAC develops from a metaplastic, premalignant columnar epithelium known as Barrett's esophagus (BE) rather than the squamous epithelium typically present in the esophagus [2–4]. BE is frequently observed in the setting of chronic gastric acid and bile reflux that results in injury and inflammation. The prevalence of Barrett's is estimated at 10–15% of patients with gastroesophageal reflux disease (GERD) [5]. Given its prevalence, its association with EAC, and the increasing incidence of EAC, understanding BE pathogenesis is an important clinical and research imperative.

**Figure 1:**
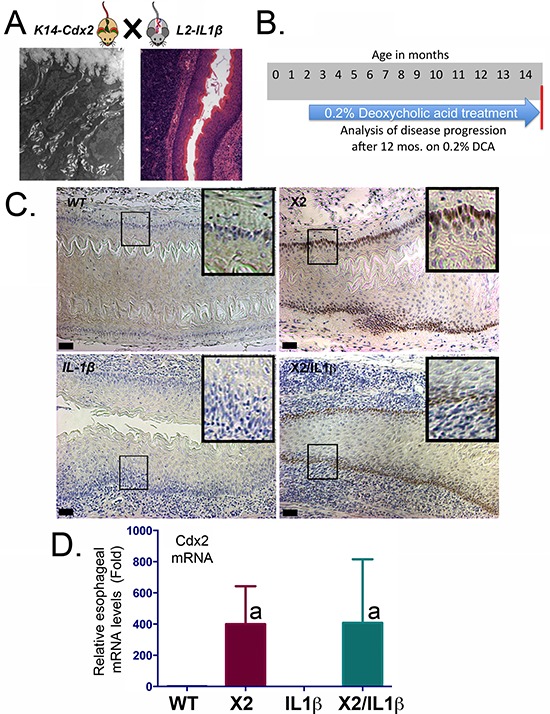
*K14-Cdx2* transgene expression is not altered by *L2-IL-1β* coexpression in *K14-Cdx2::L2-IL-1β* mice **A.** Model of the crossings to generate *K14-Cdx2::L2-IL-1β* transgenic mice. TEM image is of dilated intracellular spaces in the esophageal epithelium of *K14-Cdx2* transgenic mice. H&E image is of inflammation in the esophagus of *L2-IL-1β* transgenic mice. **B.** Experimental approach; 8-week old mice were started on 0.2% DCA in their drinking water and maintained on this for 12 months, at which time the mice were examined for disease extent. **C.** Representative Immunostaining for Cdx2 expression in esophagi of transgenic mice. (X100 magnification; black bar = 50 μm). WT: wild-type; X2: *K14-Cdx2*; IL-1β: *L2-IL-1β*; and X2/IL-1β: *K14-Cdx2::L2-IL-1β*. **D.** Cdx2 mRNA expression by Real-Time PCR analysis for Cdx2 mRNA levels for each group of mice; (*a* = significantly differs from WT and *IL-1β* controls by one-sided ANOVA and Tukey Multiple Comparisons testing, adjusted *p* < 0.047; *n* = 6).

An important focus of our research is the development of novel mouse transgenic models for BE and EAC. Previously we ectopically expressed Cdx2, an intestine-specific transcription factor commonly observed in BE, in the mouse esophagus and forestomach using the murine *Keratin 14* gene promoter [6]. Cdx2 expression was associated with altered cell morphology and ultrastructure of the esophageal epithelium. In particular we observed dilated intercellular spaces between the squamous basal cells and a compromised epithelial barrier (Figure 1A). However, the formation of a true intestinal metaplasia did not occur.

More recently, a physiologically relevant transgenic mouse model for BE and EAC was described by our group [7]. It utilized an Epstein-Barr virus L2 promoter to over-express human IL-1β in the oral cavity, esophagus, and squamous forestomach of mice. These *L2-IL-1β* mice develop a chronic [8] inflammatory esophagitis by 3 months (Figure 1A) that is followed subsequently by the development of a columnar metaplasia with intestinal features that later progresses to dysplasia and cancer. The strength of this transgenic mouse model is that in many ways it strongly phenocopies the pathogenesis of the human Barrett's esophagus as it is presently believed to occur [4, 9], with a chronic inflammatory esophagitis preceding the onset of metaplasia, followed subsequently by dysplasia and cancer. Moreover, this disease sequence is accelerated in the *L2-IL-1β* mice by the addition of bile acids, as is hypothesized for the human disease. In addition, based on histologic and molecular criteria, the columnar metaplasia which develops in the *L2-IL-1β* resembles that of human BE [7]. Lastly, the metaplasia, dysplasia and cancer arise at the squamo-columnar junction (SCJ) much as in the human disease. Together, these observations all suggest the *L2-IL-1β* mouse is an excellent animal model for human BE and EAC. However, there are limitations of this animal model. Anatomically, mice have a squamous forestomach, and therefore this metaplasia arises at the SCJ in the stomach. In addition, although the production of intestinal mucins is strongly observed and consistent with an intestinalized metaplsia, mature goblet cells are not typically seen unless the animals are treated with Notch signaling inhibitors. For this reason, the metaplasia that develops has been described as “Barrett's-like metaplasia” [7].

Given that Cdx2 is expressed in BE, is required for the intestinal phenotype [10], and that ectopic expression of Cdx2 in the esophagus induces a barrier dysfunction, we hypothesized that the *K14-Cdx2* transgene would synergize with the *L2-IL-1β* transgene and promote a more rapid progression to metaplasia and cancer. Unexpectedly, the double transgenic mice had fewer metaplastic nodules at the SCJ compared to the *L2-IL-1β* control mice. This was not due to diminished esophagitis or systemic inflammation. The reduction was due to an observed increase in apoptosis in the developing metaplasia at the SCJ of the double-transgenic mice that was not present in the single transgenic controls. Mechanistically, we provide evidence that this apoptosis is immune-mediated and increased due to significant reductions in the levels of an immune-suppressing subpopulation of immature CD11^+^Gr-1^+^ myeloid cells. These CD11^+^Gr-1^+^ cells have been implicated in promoting tumorigenesis in a number of mouse models of cancer [11–13]. We conclude this population of immature myeloid cells with immune suppressor function are critical for disease progression in the *L2-IL-1β* transgenic mouse model for BE and EAC.

## RESULTS

### Ectopic Cdx2 expression in murine esophageal epithelium does not alter the inflammatory esophagitis induced by transgenic *IL-1β* expression

To investigate for synergy between ectopic esophageal expression of the intestine-specific transcription factor Cdx2 (*K14-Cdx2*) and an animal model for Barrett's-like metaplasia, *L2-IL-1β* transgenic mice, we crossed them to yield doubly transgenic *K14-Cdx2::L2-IL-1β* mice (Figure 1A). To enhance the onset of metaplasia, we treated all mice in this study with 0.2% deoxycholic acid treatment (DCA, pH 7.0) in the drinking water beginning at 8 weeks of age and continued this treatment for 12 months total (to age 14 months, Figure 1B), as was done in the initial report describing the IL-1β transgenic model for a Barrett's-like metaplasia [7]. Histologic analysis of the esophagus confirmed Cdx2 protein expression in the basal epithelial cell population of only the *K14-Cdx2* and *K14-Cdx2::L2-IL-1β* transgenic lines but not in wild-type littermates or *L2-IL-1β* transgenic mice (Figure 1C). This indicates that prolonged DCA exposure does not itself induce Cdx2 expression in the murine esophagus. In both of the *K14-Cdx2* containing lines, Cdx2 mRNA levels induced over WT control were no different (400-fold ± 100 in *K14-Cdx2* mice vs 408-fold ± 160 in *K14-Cdx2::L2-IL-1β* mice; *n* = 6) and protein expression levels are similarly equivalent by immunohistochemistry and not affected by the *L2-IL-1β* transgene (Figure 1C and 1D).

**Figure 2:**
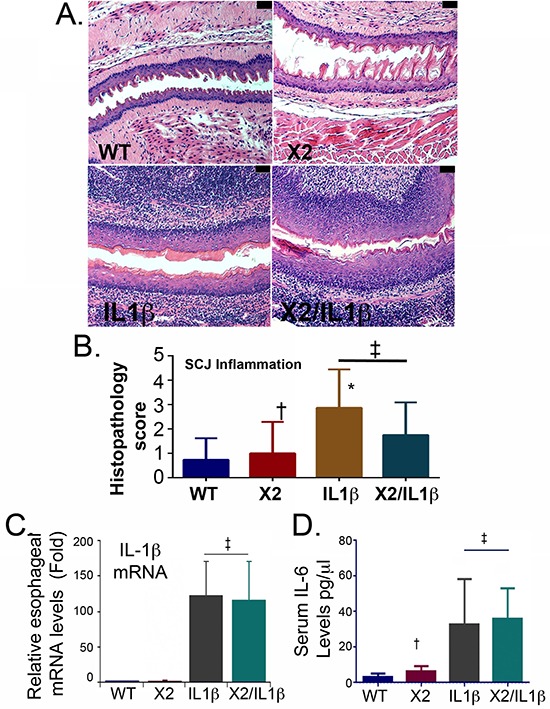
Both *L2-IL-1β and K14-CDX2::L2-IL-1β* mice demonstrate equivalent inflammatory activity **A.** Histopathologic alterations in the esophagus of *L2-IL-1β* and *K14-Cdx2::L2-IL-1β* transgenic mice (X100 magnification; black bar = 50 μm). WT: wild-type; X2: *K14-Cdx2*; IL-1β: *L2-IL-1β*; and X2/IL-1β: *K14-Cdx2::L2-IL-1β.* **B.** Score of inflammation at SCJ by observers blinded to genotype at 14 months of age. *L2-IL-1β* and *K14-Cdx2::L2-IL-1β* mice develop increased inflammation that is significant different from WT mice but not different from each other. ‡ Not significantly different, (adjusted *p* = 0.40); † not significantly different from WT control (adjusted *p* > 0.99); * significantly differs from WT mice (adjusted *p* ≤ 0.0002) by Kruskal-Wallis ANOVA and Dunn's multiple comparisons testing. **C.** Determination of esophageal IL-1β mRNA levels by qPCR analysis. Significance determined by one-sided ANOVA and Tukey's Multiple Comparison testing, *n* = 6 for each line. (‡ both significantly differ from WT control adjusted *p* < 0.01 but no significant difference between *L2-IL-1β* and *K14-CDX2/L2-IL-1β* mice). **D.** ELISA for serum levels of IL-6, *n* = 4. († not significantly different from WT mice by one-sided ANOVA and Tukey Multiple comparisons testing, adjusted *p* = 0.99; ‡ both significantly differ from WT control adjusted *p* < 0.03 but no significant difference between each other).

The *L2-IL-1β* transgene induces a brisk inflammatory infiltrate in the esophagus, oral cavity, and tongue of both *L2-IL-1β* possessing transgenic lines (Figure 2A and data not shown). At the SCJ where the metaplasia occurs, the level of inflammation is not significantly different in both IL-1β expressing transgenic lines, based on histopathology scoring by observers blinded to the genotype (Figure 2B). Levels of IL-1β mRNA in the esophagus were not altered by the *K14-Cdx2* transgene (122-fold ± 48 induction over WT controls in *IL-1β* mice against 116-fold ± 54 induction in *K14-Cdx2::L2-IL-1β* mice ; *n* = 6) (Figure 2C). Lastly, systemic inflammation induced by IL-1β expression is similarly unaffected by the *K14-Cdx2* transgene. Serum levels for IL-1β were below our ability to measure reliably in both *K14-Cdx2::L2-IL-1β* and *L2-IL-1β* mice (data not shown). However, levels of the pro-inflammatory cytokine IL-6, which is required for the *L2-IL-1β* metaplasia phenotype [7], were not measurably different in *L2-IL-1β* (33 ± 11pg/μl; *n* = 5) and *K14-Cdx2::L2-IL-1β* mice (36 ± 7pg/μl; *n* = 6) (Figure 2D). Together, these findings strongly suggest the local esophageal as well as systemic inflammatory activities are equivalent in *L2-IL-1β* and *K14-Cdx2::L2-IL-1β* mice, and that the *K14-Cdx2* transgene does not appear to affect the inflammatory response.

### Ectopic Cdx2 expression in murine esophageal and forestomach epithelium reduces metaplasia development at the squamo-columnar junction in the *IL-1β* transgenic mice

**Figure 3:**
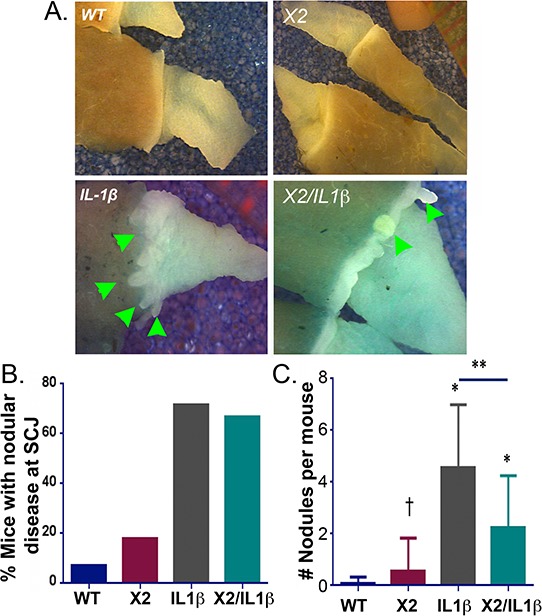
*K14-Cdx2/L2-IL-1β* TG mice metaplastic nodule numbers and nodule volume are reduced compared to *L2-IL-1β* TG mice **A.** SCJ nodular metaplasia (Green arrowhead) was present in most of *K14-Cdx2::L2-IL-1β* and *L2-IL-1β* single TG mice by 12 months of DCA treatment distributed along the SCJ of stomach. WT: wild-type; X2: *K14-Cdx2*; IL-1β: *L2-IL-1β*; and X2/IL-1β: *K14-Cdx2::L2-IL-1β.* **B.** Percentage of mice with nodular metaplasia after 12 months of DCA. WT *n* = 14; *K14-Cdx2 n* = 11; *L2-IL-1β n* = 19; *K14-Cdx2::L2-IL-1β n* = 17. **C.** Average number of nodular metaplastic lesions per mouse; WT *n* = 14; *K14-Cdx2 n* = 11; *L2-IL-1β n* = 19; *K14-Cdx2::L2-IL-1β n* = 17. Using one-way ANOVA and Tukey's Multiple comparison test ** significantly differs from each other adjusted *p* =0.0016; * Significantly differs from WT mice adjusted *p* < 0.009; † no significant difference between *K14-Cdx2* and WT mice adjusted *p* = 0.9155.

At the end of the DCA treatment period, the mice were sacrificed, and the squamo-columnar junctions (SCJ) were examined under a dissecting microscope with a dilute methylene blue stain. A prominent, nodular metaplasia was found at the SCJ in nearly 75% of the *L2-IL-1β* mice (*n* = 19), in keeping with previously reported observations ([7] and Figure 3A and 3B). In the double transgenic *K14-Cdx2::L2-IL-1β* mice, the nodular metaplasia was similarly observed in nearly 70% of mice (*n* = 17). Unexpectedly, nearly 20% of the single transgenic *K14-Cdx2* mice developed small single nodules at the SCJ (*n* = 11), much smaller than the disease noted in the *L2-IL-1β* and *K14-Cdx2::L2-IL-1β* mice. These small nodules were seen only in the *K14-Cdx2* mice receiving DCA in their drinking water (Figure 3B and data not shown).

Despite their similar disease frequencies, *K14-Cdx2::L2-IL-1β* mice appeared to have a reduced burden of nodular metaplasia compared to the *L2-IL-1β* mice (Figure 3A). To quantify this, we counted the numbers of nodules per mouse, as well as determined the volume of this metaplasia using standard tumor volume approaches. Most significantly, the *K14-Cdx2::L2-IL-1β* mice (2.2 nodules/mouse ± 2.0; *n* = 17) had half as many metaplastic nodules as the *L2-IL-1β* littermates (4.6 nodules/mouse ± 2.4; *n* = 19) (Figure 3C). There were no significant differences in the average size of the nodules in the *L2-IL-1β* and *K14-Cdx2::L2-IL-1β* mice (data not shown). However the nodules in both were significantly larger than those observed in the *K14-Cdx2* mice (data not shown). Overall, the nodule burden in the *L2-IL-1β* mice was greatest, primarily due to the increased numbers of nodules in these mice. In summary, Cdx2 co-expression with IL-1β reduced the number of nodules of Barrett's-like metaplasia observed in the *L2-IL-1β* mice treated with DCA for 12 months.

### Apoptosis is significantly increased in the Barrett's-like metaplasia of *K14-Cdx2::L2-IL-1β* mice

**Figure 4:**
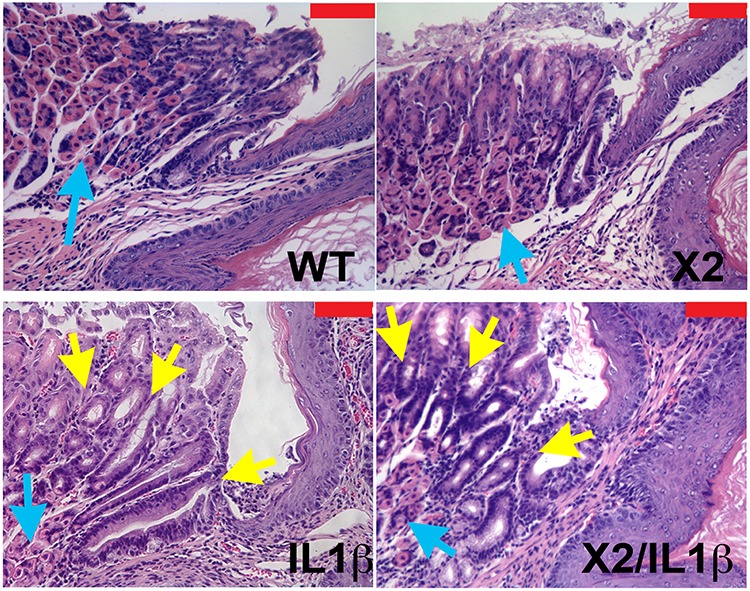
Histopathologic change in *K14-Cdx2/L2-IL-1β* double and *L2-L-1β* single transgenic mice Histopathologic changes at SCJ area in 12-month DCA-treated mice. (x200 magnification, red bar = 50 μm). Both WT and *K14-Cdx2* mice have no significant phenotypic change at the SCJ. *L2-IL-1β* and *K14-Cdx2/L2-IL-1β* mice showed more evident columnar metaplasia with displacement of the oxyntic gastric glands (blue arrow) and expansion of a glandular, columnar epithelium at the SCJ (yellow arrows). WT: wild-type; X2: *K14-Cdx2*; IL-1β: *L2-IL-1β*; and X2/IL-1β: *K14-Cdx2::L2-IL-1β*.

**Figure 5:**
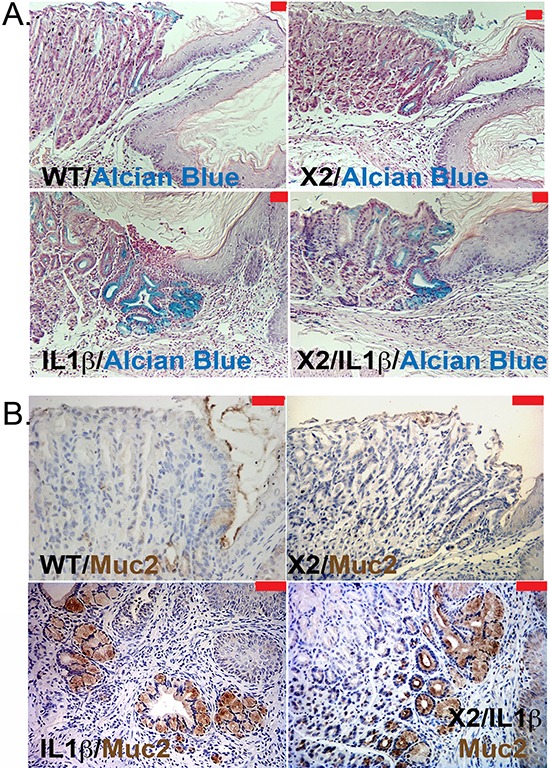
Intestinal mucin production by metaplastic glands in *L2-IL-1β* and *K14-Cdx2::L2-IL-1β* transgenic mice **A.** Representative pictures of Alcian blue staining in the SCJ metaplasia of 12-month DCA-treated mice. (X100 magnification, red bar = 50 μm). WT: wild-type; X2: *K14-Cdx2*; IL-1β: *L2-IL-1β*; and X2/IL-1β: *K14-Cdx2::L2-IL-1β.* **B.** Representative immunohistochemistry for Muc2 expression (brown stain) in the SCJ metaplasia from similar mice (red bar = 50 μm).

To understand better what the effects of Cdx2 co-expression are on the *L2-IL-1β* mouse phenotype, we next examined the metaplasia that develops in the *K14-Cdx2::L2-IL-1β* mice. The metaplasia which arises in *L2-IL-1β* mice has been previously-described as Barrett's-like based on several criteria, the most importantly being 1) the induction of intestinal mucin-producing cells (but not classic goblet cells), 2) the disease arising in the setting of chronic inflammation at the SCJ (as occurs in the human disease), and 3) a gene expression pattern which significantly overlaps with the human BE disease [7]. As in this published description, there is an expansion of a glandular, columnar epithelium at the SCJ and displacement of the oxyntic gastric glands distally in both the *L2-IL-1β* and *K14-Cdx2::L2-IL-1β* mice (Figure 4). Histopathologically, this metaplasia was present in both the *L2-IL-1β* and *K14-Cdx2::L2-IL-1β* mice but not the WT and *K14-Cdx2* littermates. In both *L2-IL-1β* and *K14-Cdx2::L2-IL-1β* mice, the metaplastic cells express intestinal mucins, as evidenced by positive staining with Alcian blue and Muc2 (Figures 5A and 5B). Moreover, consistent with the published report, we can demonstrate increased mRNA expression of the Barrett's esophagus associated genes Cckbr, Tff2, and Krt19 (data not shown). Together these findings confirm the previously published description of the *L2-IL-1β* mouse and establish that *K14-Cdx2* co-expression does not significantly alter the phenotype of metaplasia which develops in *K14-Cdx2::L2-IL-1β* mice.

**Figure 6:**
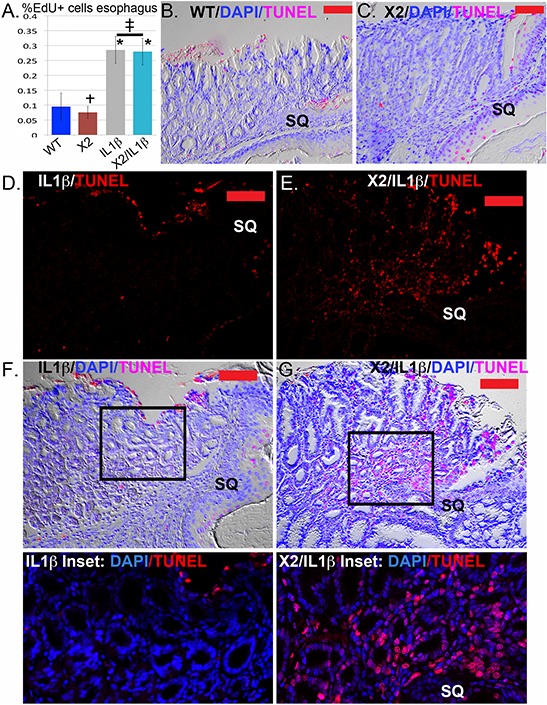
Apoptosis but not cell proliferation is significantly altered in the SCJ metaplasia *K14-Cdx2::L2-IL-1β* transgenic mice **A.** Average counts of EdU incorporation in the esophagi of transgenic and control mice. EdU was detected by Alexa Fluor 594 azide (red) and cell nuclei were stained with DAPI (blue). At least 300 cells per mouse were counted. *L2-IL-1β* over-expression promotes cell proliferation in esophageal epithelium. * Significantly differs from control WT mice by one-sided ANOVA and Tukey's Multiple comparison test adjusted *p* < 0.043. ‡ no significant difference between double and single *L2-IL-1β* TG mice adjusted *p* = 0.9996; † no significant difference between *K14-Cdx2* and WT mice, adjusted *p* = 0.9932. *n* = 15 mice WT, *n* = 8 mice *K14-Cdx2*, *n* = 15 mice *L2-IL-1β*, and *n* = 10 *K14-Cdx2::L2-IL-1β* mice. **B.** and **C.** Representative imaging of combined nuclear (DAPI-blue), TUNEL (Tetramethylrhodamine –red) and differential interference contrast microscopy (Nomarski) of the SCJ of (B) WT and (C) *K14-Cdx2* mice. (x200, red bar = 50 μm). **D.** and **E.** Representative imaging TUNEL (Tetramethylrhodamine –red) staining of the SCJ metaplasia of (D) *L2-IL-1β* and (E) *K14-Cdx2::L2-IL-1β* mice. There is a highly significant increase in TUNEL labeling of the nuclei of the glandular epithelial cells. (x200, red bar = 50 μm). **F.** and **G.** Representative imaging of combined nuclear (DAPI-blue), TUNEL (Tetramethylrhodamine –red) and differential interference contrast microscopy (Nomarski) of the SCJ metaplasia of (F) *IL-1β* and (G) *K14-Cdx2::L2-IL-1β* mice. (×200, red bar = 50 μm). Inset: ×400 magnification of SCJ metaplasia with only nuclear (DAPI-blue) and TUNEL (Tetramethylrhodamine –red) staining illustrate the localization of TUNEL staining to nuclei, including the glandular epithelium of the metaplasia.

We next considered other mechanisms by which Cdx2 co-expression in the squamous epithelium reduced the development of the nodular metaplasia at the SCJ. Cdx2 is a transcription factor with tumor-suppressor activity in the intestine, possibly mediated by inhibitory effects on cell proliferation, but it is also reported to have tumorigenic activity by repressing cell apoptosis [6, 14, 15]. In the mouse esophagus, IL-1β expression increased cell proliferation equally, as demonstrated by incorporation of the DNA analogue EdU in the Cdx2 non-expressing (0.29 ± 0.06%EdU+nuclei; *n* = 15 mice) and expressing (0.28 ± 0.07%EdU+nuclei; *n* = 10 mice) littermates (Figure 6A). At the SCJ, there was no significant effect of either transgene or DCA treatment on SCJ metaplasia EdU incorporation (data not shown), suggesting the observed reduction in metaplastic development in *K14-Cdx2::L2-IL-1β* mice was not due to changes in cell proliferation either in the esophagus or at the SCJ.

As an increase in apoptosis in the *K14-Cdx2::L2-IL-1β* mice could be an explanation, we examined apoptosis rates by Caspase 3 immunohistochemistry (IHC) and TUNEL staining. In the esophagus, there was no evidence that the *L2-IL-1β* or *K14-Cdx2* transgenes alone altered cellular apoptosis by either technique (Figure 6B and 6C and data not shown). Similarly, there was no significant apoptosis noted in the SCJ metaplasia in *L2-IL-1β* transgenic mice (Figure 6D and 6F and F-inset). However, in the *K14-Cdx2::L2-IL-1β* mice, there was a noticeable increase in apoptosis demonstrated by both approaches (Figure 6E and 6G and G-inset). The TUNEL staining in the *K14-Cdx2::L2-IL-1β* mice is localized to nuclei. This is most evident in the inset, and includes many nuclei from the metaplastic glandular epithelium (Figure 6E, 6G, and 6G-inset). In summary, the *K14-Cdx2* transgene limits the formation of the BE like metaplasia in *L2-IL-1β* transgenic mice by increasing apoptosis in the developing SCJ metaplasia.

### Myeloid cell associated genes are diminished in the metaplastic nodules of *K14-Cdx2::L2-IL-1β* mice

**Figure 7:**
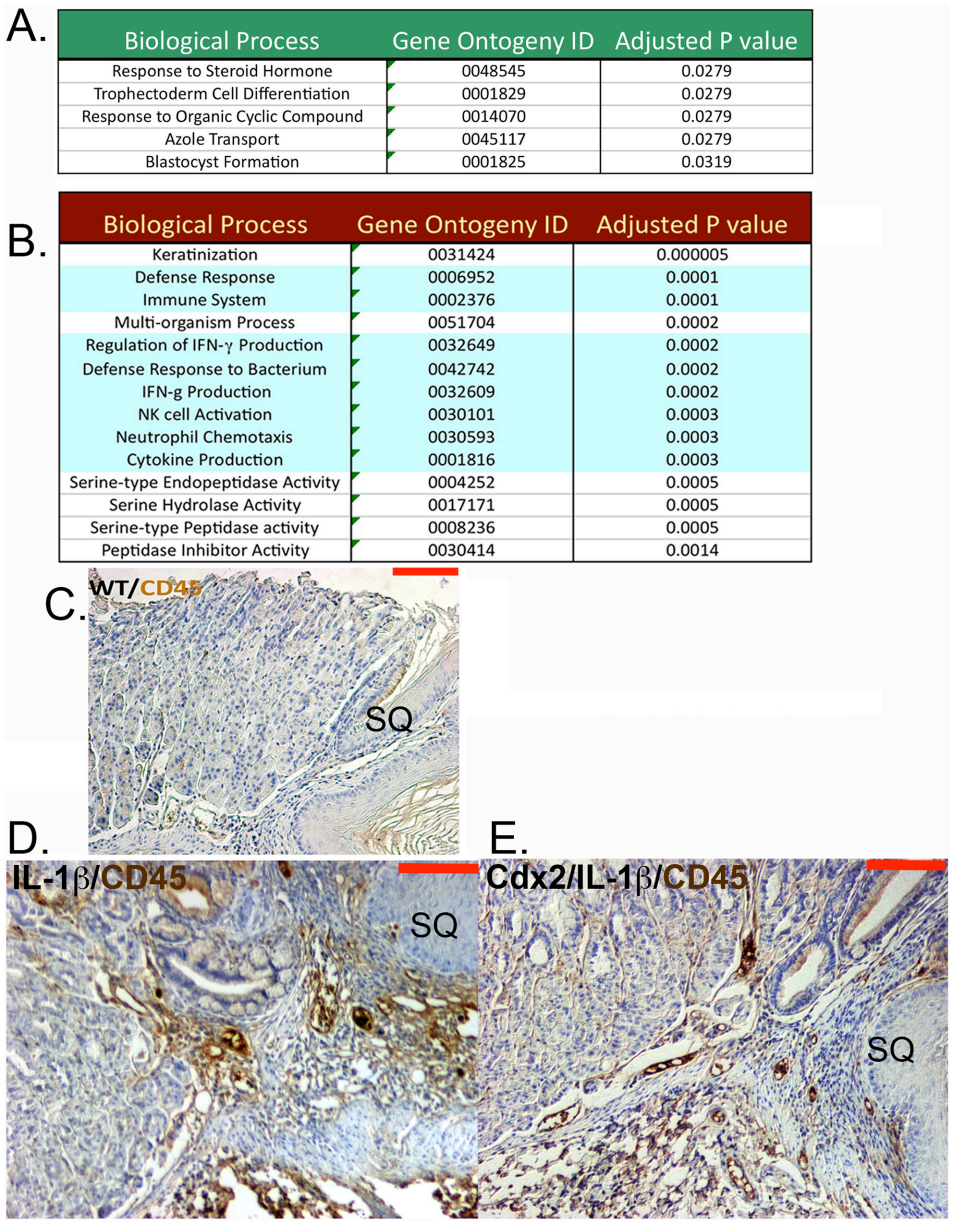
Cdx2 overexpression in *L2-IL-1β* transgenic mice leads to downregulated myeloid cell genes in the SCJ metaplastic nodules but no effect on CD45+ cell infiltration An Affymetrix microarray analysis of gene expression in the SCJ nodules from *K14-Cdx2::L2-IL-1β* mice and *L2-IL-1β* mice was performed. **A.** Green box: A partial list of the gene function annotation using DAVID bioinformatic resources of the 47 genes whose expression is increased in *K14-Cdx2::L2-IL-1β* mice with a *p* < 0.05. **B.** Red Box: A partial list of the gene ontogeny analysis of the 152 genes diminished in *K14-Cdx2::L2-IL-1β* mice compared to *L2-IL-1β* mice with a *p* < 0.05. Green shading highlights immune or inflammation associated processes. **C–E.** Immunohistochemistry for CD45+ cells at the SCJ from 12-month DCA treated mice. (C) Non-transgenic WT mice, (D) *L2-IL-1β* mice, and (E) *K14-Cdx2::L2-IL-1β* mice (X200 magnification, red bar = 50 μm).

In order to determine why cell apoptosis is increased in the developing metaplasia of the double transgenic mice, we performed an Affymetrix microarray analysis of gene expression differences. We compared gene expression in the metaplastic nodules from *L2-IL-1β* and *K14-Cdx2::L2-IL-1β* mice. We identified 199 genes whose expression differed by 2-fold or more and had less than a 10% false discovery rate (Table 1 and Supplementary Table S1). Only 47 of the 199 differentially expressed genes had increased levels in the *K14-Cdx2::L2-IL-1β* mice. The two most strongly induced were Syncollin (*Sync*) and Cadherin related family member 5 (*Cadhr5*), both of which are expressed in the intestine and one, Cadhr5, is a known transcriptional target of Cdx2 [16]. A gene function annotation of these 47 genes using DAVID bioinformatic resources [17] found only a few weak associations (*p* values between 0.02 and 0.05) with early developmental processes (Figure 7A).

**Table 1:** Abbreviated list of genes whose mRNA levels were significantly different

Gene Symbol	Gene Name	Fold-Change	*p*-value
Sycn	Syncollin	13.5724	0.00640984
Cdhr5	Cadherin-related family member 5	7.18268	0.0117104
Cdx2	Caudal type homeobox 2	6.622	0.000418314
Ifi27l2b	Interferon, alpha-inducible protein 27 like 2B	6.07215	0.00849458
Far2	Fatty acyl CoA reductase 2	5.33113	0.00304483
Mir145	mIR-145	3.99754	0.0115672
Lce1k	Late cornified envelope 1K	3.73066	0.0275127
Krt79	Keratin 79	3.71593	0.0108772
P2ry2	Purinergic receptor P2Y, G-protein coupled 2	−2.08968	0.00444745
Pglyrp1	Peptidoglycan recognition protein 1	−2.12096	0.0136792
Il23a	Interleukin 23, alpha subunit p19	−2.13829	0.00144615
Gzmb	Granzyme B	−2.14237	0.00477608
Selplg	Selectin, platelet (p-selectin) ligand	−2.1509	0.010656
Il27ra	Interleukin 27 receptor, alpha	−2.18232	0.000621474
Ptpn22	Protein tyrosine phosphatase, non-receptor type 22 (lymphoid)	−2.19618	0.00301512
Stap1	Signal transducing adaptor family member 1	−2.24255	0.0120524
Blk	B lymphoid kinase	−2.29922	0.00536239
Il2rb	Interleukin 2 receptor, beta chain	−2.33622	3.26E-05
Cd3e	CD3 antigen, epsilon polypeptide	−2.44474	0.0169952
Nox1	NADPH oxidase 1	−2.61431	0.00624271
Mmp9	Matrix metallopeptidase 9	−2.62158	0.0227285
Clec4e	C-type lectin domain family 4, member e	−2.67522	0.00210464
Defb14	Defensin beta 14	−2.72681	0.0271445
Lcn2	Lipocalin 2	−2.76479	0.00137978
Il17c	Iinterleukin 17C	−2.77006	0.050003
Csf3r	Colony stimulating factor 3 receptor (granulocyte)	−2.8235	0.0185792
Il17a	Interleukin 17A	−2.87594	0.00287537
Cxcr6	Chemokine (C-X-C motif) receptor 6	−3.08408	0.0097923
Pglyrp4	Peptidoglycan recognition protein 4	−3.14416	0.00447996
Ifitm1	Interferon induced transmembrane protein 1	−3.21242	0.00218518
S100a8	S100 calcium binding protein A8 (calgranulin A)	−3.28772	0.000840821
S100a9	S100 calcium binding protein A9 (calgranulin B)	−3.73631	0.000837659
Defb3	Defensin beta 3	−3.94574	0.000132201
Wfdc12	WAP four-disulfide core domain 12	−3.96555	0.0344597
Olfm4	Olfactomedin 4	−3.96623	0.0137842
Ifi202b	Interferon activated gene 202B	−5.07291	0.00164929
Akr1c18	aldo-keto reductase family 1, member C18	−10.1761	0.0246727
Uox	Urate oxidase	−14.9333	0.00691962

Determined by Affymetrix microarray analysis of SCJ metaplasia isolated from *K14-Cdx2::L2-IL-1β* and *L2-IL-1β*. *n*=3 biological replicates for each, with *p*≤0.05 for each listed gene.

A gene ontogeny analysis of genes whose expression was diminished in the double transgenic mice revealed a number of defense and immune response pathways significantly associated (*p* values ≥ 0.0005) with the 152-gene list (Figure 7B). In particular, there were several genes reduced whose products are associated with immature myeloid cells, including IL-17a, IL-17c, IL-23a, S100A8, S100A9, and Csf3r (Colony stimulating factor 3 receptor-granulocytes), as well as reductions in Granzyme B and a number of serine proteases suggestive of cytotoxic T cells (Table 1). We had previously established that the inflammation in the esophagus and systemic IL-6 levels were no different between the two transgenic lines. To explore whether the inflammatory infiltrate at the SCJ was diminished in *K14-Cdx2::L2-IL-1β* mice, we stained for CD45+ infiltrating inflammatory cells. We found that both *L2-IL-1β* and *K14-Cdx2::L2-IL-1β* mice maintained a brisk CD45+ immune cell infiltration at the SCJ junction as compared to normal WT littermates (Figure 7C, 7D, and 7E). Therefore, while the microarray results suggest there may be significant alterations in a subset of myeloid cells in the developing metaplasia, the broader increased inflammatory response is intact in the *K14-Cdx2::L2-IL-1β* mice.

### Immature myeloid cells are diminished in the *K14-Cdx2::L2-IL-1β* mice

**Figure 8:**
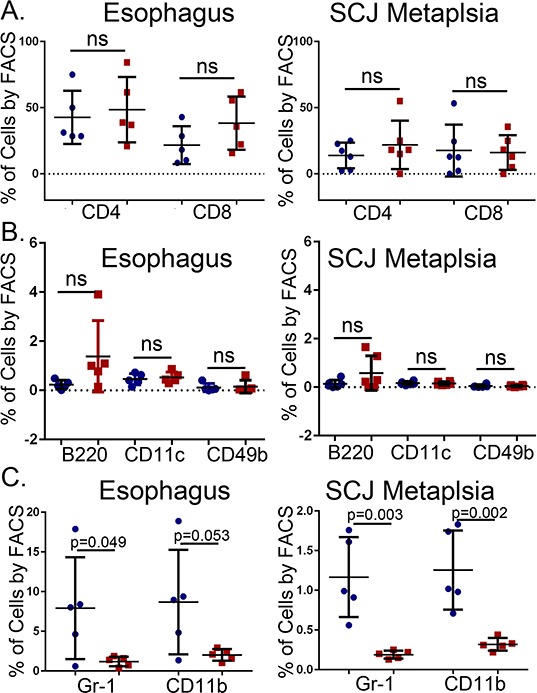
No changes in the numbers of infiltrating T-cells, B-cells, dendritic cells, and natural killer (NK) cells but a reduction in Gr-1+ and CD11b+ cells in the esophagus and SCJ metaplasia of *K14-Cdx2::L2-IL-1β* transgenic mice Immune cell subpopulations in the esophagus and the nodular metaplasia were examined by flow cytometry. We quantified levels of T cells (CD4^+^, CD8^+^), B cells (B220^+^), dendritic cells (CD11c^+^), natural killer (NK) cells (CD49b^+^), granulocytes (Gr-1^+^) and monocytes (CD11b^+^). All cells were 7AAD^−^ and CD45^+^ in addition to the specific cell surface marker. **A–B.** Graphical representation of multiple FACS measures from 5 mice for each cell type. Blue circle: *L2-IL-1β* mice. Red squares: *K14-Cdx2::L2-IL-1β* transgenic mice. The flow cytometry percentages were Log transformed for statistical analysis (*p* values determined by unpaired two-tailed *T*-test, ns : non-significant, *p* > 0.05).

To gain a clearer understanding of how the *K14-Cdx2* transgene is altering the nature of the inflammatory response in the *L2-IL-1β* mice, we carefully characterized immune cell populations infiltrating the esophagus and SCJ metaplasia by flow cytometry. Levels of T-cells (CD4, CD8), B-cells (B220), dendritic cells (CD11c), natural killer (NK) cells (CD49b) were not altered by the *K14-Cdx2* transgene in either the SCJ metaplasia or esophagus (Figure 8A and 8B; *n* = 5 mice for each genotype, *p* > 0.05).

**Figure 9:**
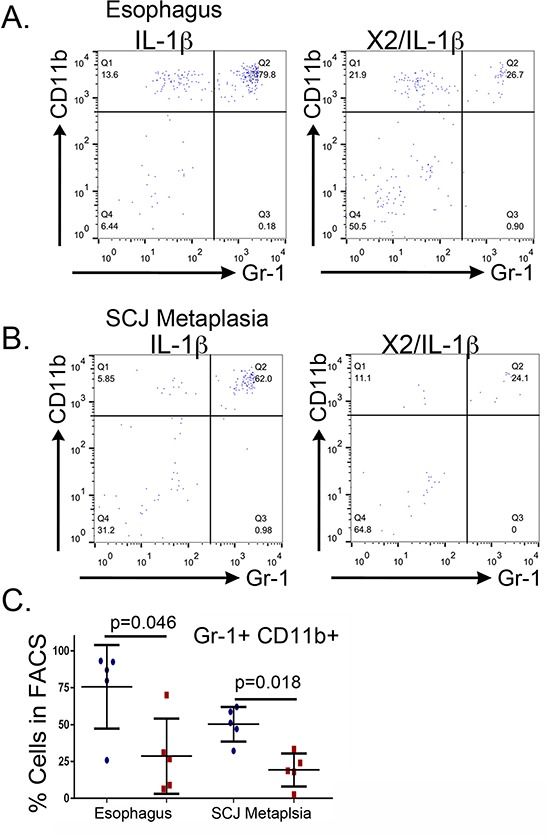
Reduced metaplasia is associated with depletion of *CD11b^+^Gr-1^+^* double-positive cells in the esophagus and SCJ metaplasia of *K14-Cdx2::L2-IL-1β* transgenic mice We quantified levels of the double-positive CD11b^+^Gr-1^+^ in the esophagus and SCJ metaplasia. All cells were 7AAD^−^and CD45^+^ in addition to the specific cell surface marker. **A, B.** A sample FACS experiment demonstrating the significant reduction in CD11b^+^ Gr-1^+^ double positive cells within the infiltrating CD45^+^ population of the (B) esophagi and **C.** SCJ metaplasia of *K14-Cdx2::L2-IL-1β* mice when compared to *L2-IL-1β* mice, one of 5 experiments shown. (C) Graphical representation of multiple FACS measures from the esophagus and SCJ metaplasia of 3 to 5 *L2-IL-1β* and *K14-Cdx2::L2-IL-1β* mice each. Blue circle: *L2-IL-1β* mice. Red squares: *K14-Cdx2::L2-IL-1β* transgenic mice. The flow cytometry percentages were Log transformed for statistical analysis (*p* values determined by unpaired two-tailed *T*-test, ns : non-significant, *p* < 0.05) and graphing.

There were greater changes in other subpopulations. Cell surface markers for granulocytes (Gr-1) and monocytes (CD11b) appeared to be significantly diminished in the *K14-Cdx2::L2-IL-1β* mice, by 4 to 8-fold in both the esophagus (*p* = 0.049 and *p* = 0.053, respectively *n* = 5 mice each genotype) and the SCJ metaplasia (*p* = 0.003 and *p* = 0.002, respectively, *n* = 5 mice each genotype) (Figure 8C), suggesting this effect was systemic and not confined to the SCJ. Both of these markers can also be co-expressed on immature myeloid cell populations [12]. Surprisingly, the majority of CD11b+ and Gr-1+ cells were in fact double positive, consistent with these cells representing an immature myeloid population (Figure 9). This population was described previously in the *L2-IL-1β* mice [7], but their importance for the development of the SCJ metaplasia was not evident. We do not know when these CD11b^+^Gr-1^+^ cells are first present but we have detected them at 9 months (data not shown) and Quante et. al. reported them in 6 month old mice [7], before the onset of the SCJ metaplasia.

**Figure 10:**
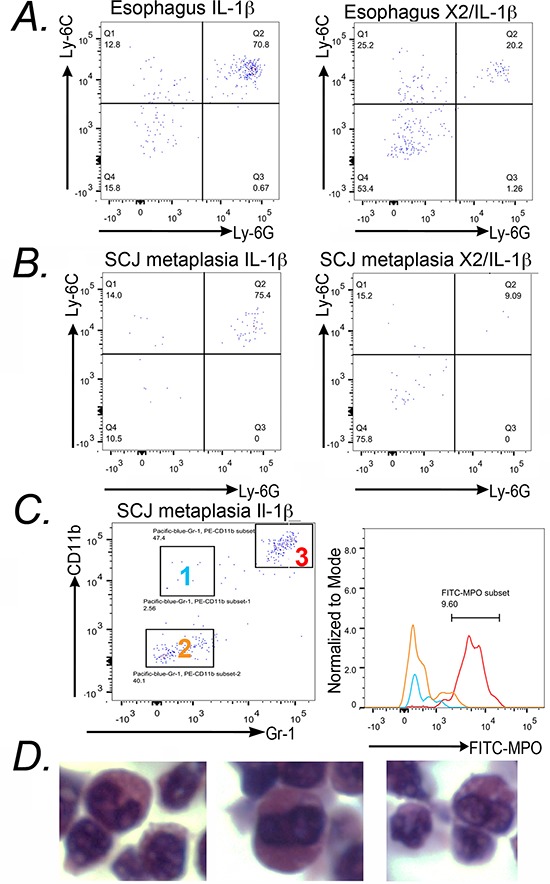
CD11b+/Gr-1+ cells from L2-IL-1β mice express cell surface and intracellular markers that identify them as immature granulocytes **A.** 7AAD^−^CD45^+^CD11b^+^ cells from the esophagus were stained with anti-Ly-6G and anti-Ly-6C for FACS analysis. The majority of CD11b+ cells are positive for both antigens. *n* = 3 mice studied. **B.** Similar findings in the FACS analysis for Ly-6G and Ly-6C expression in 7AAD^−^CD45^+^CD11b^+^ cells isolated from the SCJ metaplasia of *L2-IL-1β* and *K14-Cdx2::L2-IL-1β* mice. As before, the majority of CD11b+ cells are Ly-6G^+^/Ly-6C^+^ and the levels of these double-positive cells are diminished in the SCJ metaplasia of *K14-Cdx2::L2-IL-1β* mice. *n* = 3 mice studied. **C.** Dot-plots from FACS analysis indicated 3 subpopulations of CD45^+^ cells with anti-CD11b and anti-Gr-1 staining. Relative intracellular staining for MPO in each subset of cells was determined and plotted on a histogram. Plots represent the intracellular staining of MPO in Group 1 (CD45^+^CD11b^+^Gr-1^−^ cells: Blue line), Group 2 (CD45^+^CD11b^−^Gr-1^−^: Yellow line) and Group 3 (CD45^+^CD11b^+^Gr-1^+^ : Red line). *n* = 3 mice studied. **D.** Hematoxylin and Eosin staining of pelleted 7AAD^−^CD45^+^Gr-1^+^CD11b^+^ cells isolated from an *L-2-IL-1β* transgenic mouse.

CD11b^+^Gr-1^+^ immature myeloid cells originate in the bone marrow in response to factors secreted by tissues and tumors. They have been implicated in enhancing tumor growth in the breast, colon, and pancreatic cancers [11, 12], but have not been described in precancerous conditions previously. These immature myeloid cells can enhance tumorigenesis by several mechanisms, including inhibition of tumor cell immune surveillance (mediated by cytotoxic T-cells), enhancement of angiogenesis, and the overall promotion of tumor cell survival [11, 12]. Immature myeloid cells can belong to either the monocytic or granulocytic lineages [18]. To determine which lineage these cells belonged we examined for the expression of Ly-6C and Ly-6G in CD11b+ cells. Monocytic lineages are Ly-6G^−^ Ly-6C^Hi^ whereas granulocytic lineages are Ly-6G^+^ and Ly-6C^Lo/+^. We found the CD45^+^CD11b^+^ cells from esophagus and SCJ metaplasia of the *L2-IL-1β* mice were Ly-6G^+^ and Ly-6C^+^ (Figure 10A, 10B), which is consistent with a granulocytic lineage as previously described [12]. Moreover, these cells are largely lost in the double transgenic *K14-Cdx2/L2-IL-1β* mice (*n* = 3 mice for each genotype) (Figure 10A, 10B). As an additional confirmation of their lineage, we performed intracellular staining for myeloperoxidase (MPO), a lysosomal enzyme abundantly expressed in neutrophil granulocytes. CD45^+^CD11b^+^Gr-1^+^ cells from the SCJ metaplasia strongly express MPO, in contrast to the CD45^+^CD11b^+^Gr-1^−^ and CD45^+^CD11b^−^Gr-1^−^ populations from the same tissue (Figure 10C). Lastly, immature granulocytes are also known as band cells due to their distinct densely staining and unsegmented nucleus [19]. We isolated CD45^+^CD11b^+^Gr-1^+^ cells by FACS, pelleted, fixed, sectioned and stained them for histologic analysis. Morphologically these cells possess the classic prominent, unsegmented nuclei of band cells (Figure 10D), further establishing their identity as an immature granulocytic cell population.

**Figure 11:**
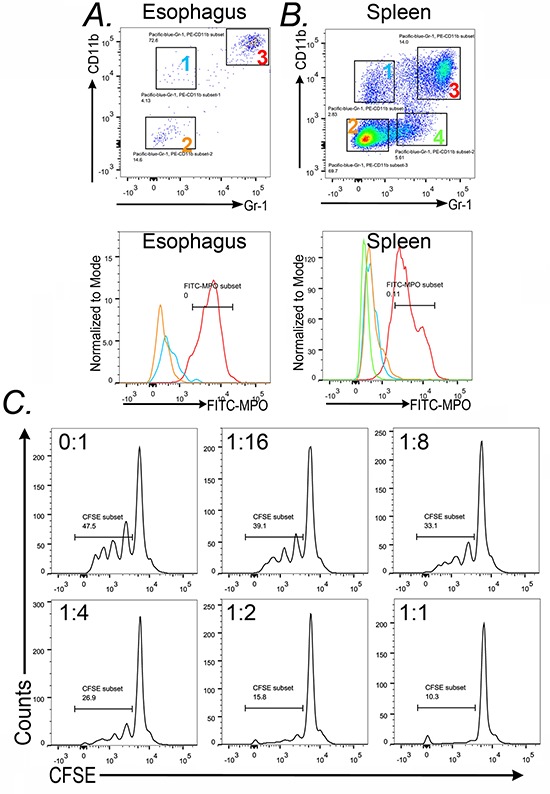
CD11b+Gr-1+ from the spleen and esophagus of *L-2-IL-1β* mice also express MPO+ (Myeloperoxidase) and can suppress CD4+ T-cell proliferation Dot-plots from FACS analysis of **A.** esophagi and **B.** spleen of *L2-IL-1β* mice illustrate the gating of the subpopulations analyzed. In the esophagus there are three populations-group 1 (blue): Gr-1^−^ CD11b^+^, group 2 (yellow): Gr-1−CD11b− and group 3 (Red): CD11b^+^Gr-1^+^. FACS analysis for spleen tissue indicated 4 subpopulations including the same three as in the esophagus plus Group 4 (green) Gr-1^+^ CD11b^−^. Histogram plots represent the expression of MPO on the gated subsets Group 1 (Blue), Group 2 (Yellow), Group 3 (Red), and Group 4 (green). **C.** Splenocytes from wild-type C57Bl6 mice were prelabeled with CSFE and then cultured with anti-CD3/CD28 beads and increasing numbers of Gr-1^+^CD11b^+^ cells from *L2-IL-1b* transgenic mice. Ratio progressed from 0 (CD11b^+^Gr-1^+^ cells) : 1 (splenocytes) to as high as 1:1. CD4^+^ cell proliferation was determined by measuring cellular CFSE levels in CD3^+^/CD4^+^ cells after 72 hours. Results from one of four experiments is shown.

Two key properties of the tumor-promoting CD45^+^CD11b^+^Gr-1^+^ cell population is that they emerge from the bone marrow, and therefore are a systemic response and not tissue-specific, and that they possess immune-suppressor properties (specifically T-cell suppression) [12, 18]. To determine if these cells exist beyond the esophagus and SCJ metaplasia, we isolated CD45^+^CD11b^+^Gr-1^+^ cells from the esophagus and the spleen and again assayed for intracellular MPO levels. We observed abundant MPO protein in the CD45^+^CD11b^+^Gr-1^+^ cell population from both organs, and little MPO in the other control cell populations (Figure 11A and 11B), suggesting they are identical cell types. Moreover, the presence of these cells in the spleen indicates this is a systemic response, not one localized to the esophagus and SCJ metaplasia.

To assay for immune suppressor function in our CD11b^+^Gr-1^+^ cells, we performed an *in-vitro* T cell suppression assay. The assay measures the ability of CD45^+^CD11b^+^Gr-1^+^ cells to suppress CD4^+^ T-cell proliferation in response to CD3/CD28 costimulation [20]. As this assay requires significant numbers of CD45^+^CD11b^+^Gr-1^+^ cells, they were isolated from the spleens of *L2-IL-1β* mice rather than the esophagus or SCJ metaplasia. As can be observed, increasing the ratio of the CD45^+^CD11b^+^Gr-1^+^ cells to CD4^+^ T-cells led to a significant decrease in the proliferative response of the T-cells to costimulation by CD3/CD28 beads (Figure 11C). Together, these observations suggest that the CD45^+^CD11b^+^Gr-1^+^ cells, which are significantly reduced in the *K14-Cdx2/L2-IL-1β* mice, are immature granulocytes with an immune-suppressor phenotype. Given the association of CD11b^+^Gr-1^+^ cells with increased tumor growth in a number of mouse models of cancer, it raises the possibility that these cells may also contribute to metaplasia formation and expansion in the *L2-IL-1β* mouse model of Barrett's-like metaplasia.

### CD8^+^ cells are required for the increased apoptosis in the SCJ metaplasia of the *K14-Cdx2/L2-IL-1β* mice

**Figure 12:**
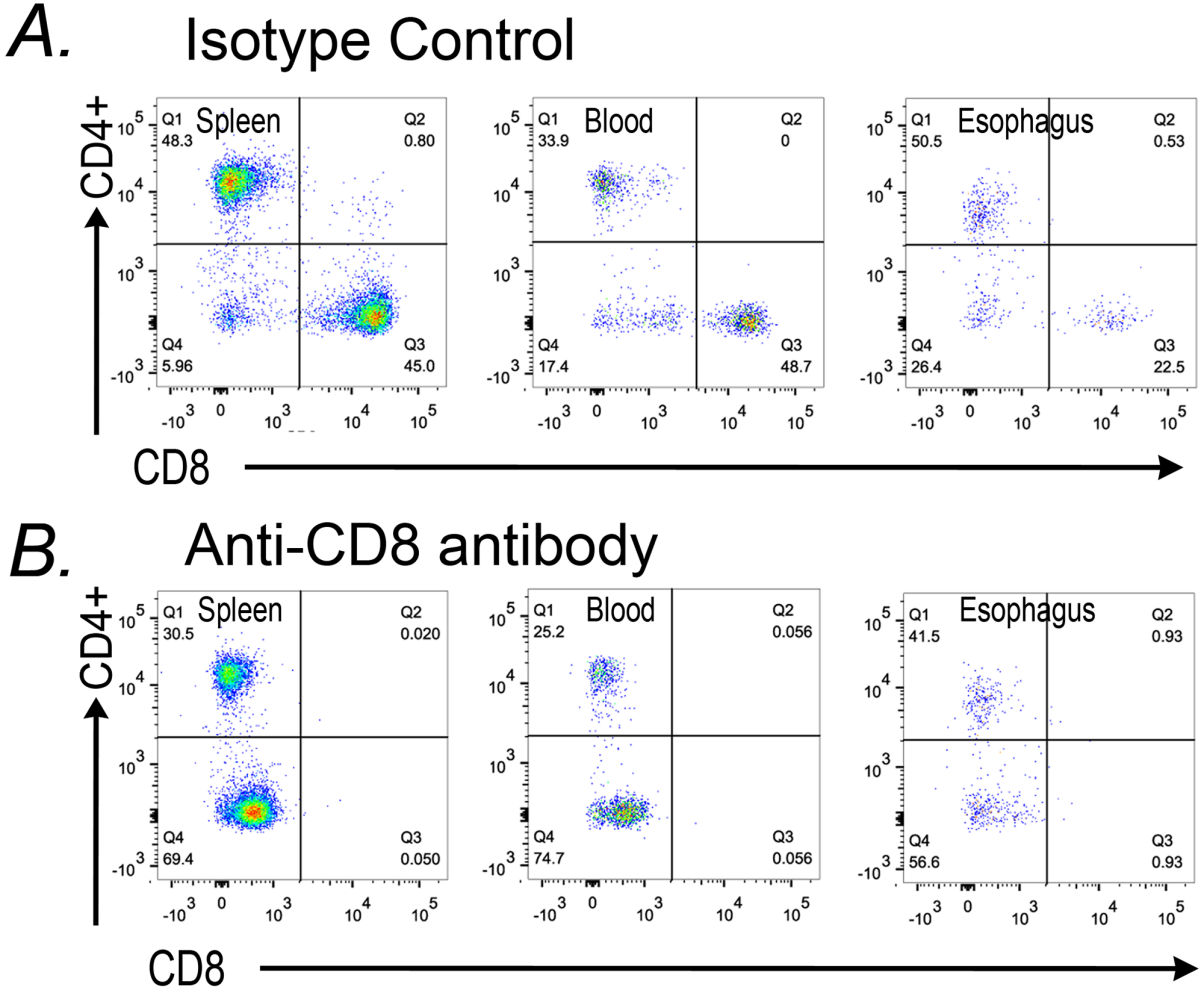
Antibody-mediated knockdown of CD8+ cells in *K14-Cdx2/L2-IL-1β* double transgenic mice *K14-Cdx2::L2-IL-1β* mice after 12 months of DCA treatment were subject to three injections with an anti-CD8 antibody or an isotype antibody control. Dot-plots from FACS analysis for CD45^+^CD8^+^ cells in the spleen, blood, and esophagus of **A.** isotype control and **B.** anti-CD8 antibody treated *K14-Cdx2::L2-IL-1β* mice. There was significant reduction in CD45^+^CD 8^+^ cells in all three tissues without an appreciable effect on CD4^+^ T-cells in those same tissues. *n* = 3 *K14-Cdx2::L2-IL-1β* mice for each condition, 6 mice total.

Studies using both mouse cancer models and human subject samples have indicated that increased levels of immature myeloid cells can suppress normal CD8^+^ cytotoxic T-cell responses and thereby enhance tumor growth [11, 12, 20]. To determine if CD8^+^ T-cells are involved in the induction of apoptosis observed in the metaplasia of *K14-Cdx2::L2-IL-1b* mice, we targeted CD8^+^ cells using an antibody. 14 month-old *K14-Cdx2::L2-IL-1b* mice were injected with 200 μg of anti-mouse CD8 antibody or an isotype control antibody on days 1, 3, and 5 and sacrificed on day 6. FACS analysis of immune cell populations in the spleen, blood and esophagus demonstrated a nearly complete absence of CD8^+^ cells in the mice receiving the anti-CD8 antibody and not those mice receiving the isotype control (Figure 12). Moreover, CD4^+^ T cell levels were not affected by either treatment, attesting to the specificity of the ablation.

**Figure 13:**
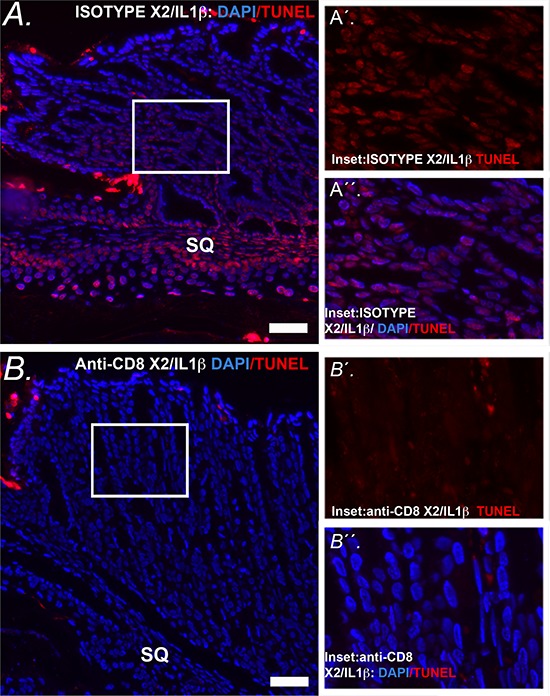
Apoptosis at the SCJ in *K14-Cdx2::L2-IL-1β* mice is lost with knockdown of CD8+ cells **A.** Representative image of combined nuclear (DAPI-blue) and TUNEL (Tetramethylrhodamine –red) at the SCJ of isotype treated *K14-Cdx2::L2-IL-1β* mice. (x200, white bar = 50 μm) SQ = squamous forestomach. **A′ inset**. Representative higher-power imaging for TUNEL staining of the apoptotic columnar metaplasia at the SCJ. **A′′ inset.** similar higher-power imaging for nuclei (DAPI-blue) and TUNEL (Tetramethylrhodamine –red). There is a highly significant TUNEL labeling of the nuclei of the glandular epithelial cells of isotype treated *K14-Cdx2::L2-IL-1β* mice *n* = 3 *K14-Cdx2::L2-IL-1β* mice for each treatment. **B.** Representative imaging of combined nuclear (DAPI-blue) and TUNEL (Tetramethylrhodamine –red) of the SCJ sections from a mouse treated with the anti-CD8 antibody demonstrating significantly reduced TUNEL+ nuclei in both the squamous and glandular compartments. (x200, white bar = 50 μm) SQ = squamous forestomach. **B′ inset.** Representative higher-power imaging demonstrating little TUNEL staining of the columnar metaplasia at the SCJ. **B′′ inset.** similar higher-power imaging for nuclei (DAPI-blue) and TUNEL (Tetramethylrhodamine –red) revealing little TUNEL labeling of the nuclei of anti-CD8 antibody treated *K14-Cdx2::L2-IL-1β* mice *n* = 3 *K14-Cdx2::L2-IL-1β* mice for each treatment.

We then assessed the SCJ metaplasia for histologic changes with the CD8^+^ cell knockdown. Since the CD8^+^ cell knockdown was a short duration, we did not observe significant changes in metaplasia abundance or morphology (data not shown). Significantly, however, there was a near complete loss of the apoptosis previously observed at the SCJ in the *K14-Cdx2::L2-IL-1b* mice (Figure 13). Under closer examination, it can be observed that the apoptosis is reduced in the squamous forestomach as well as the adjacent SCJ metaplasia (Figure 13 inset). We conclude that the elevated apoptosis in the squamous forestomach and SCJ metaplasia of the *K14-Cdx2::L2-IL-1b* mice is due to an immune-mediated and CD8+ T-cell dependent mechanism in the *L2-IL-1β* transgenic mouse model.

### *K14-Cdx2::L2-IL-1b* mice have diminished expression of known regulators of CD11b^+^Gr-1^+^ cell development

**Figure 14:**
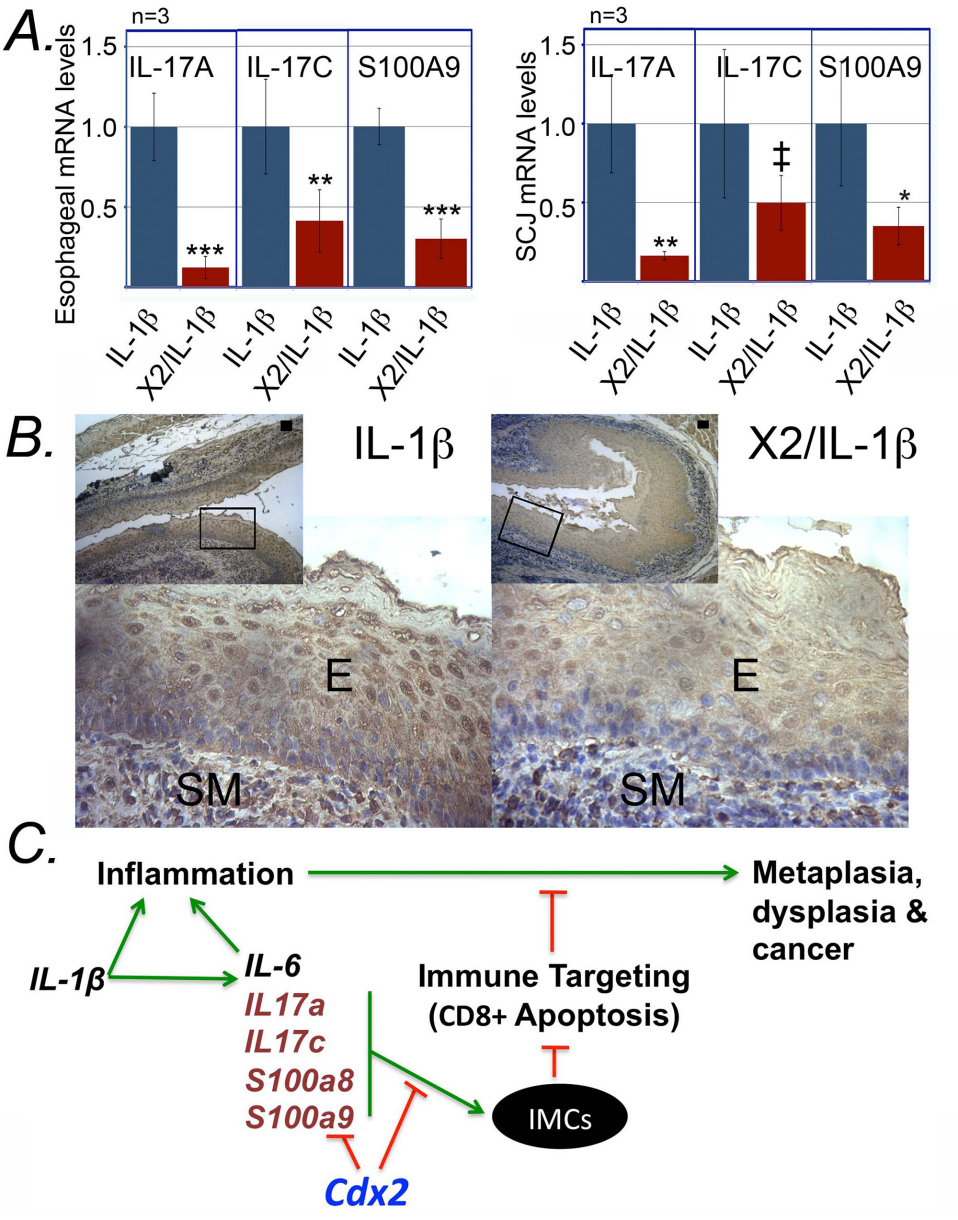
*K14-Cdx2::L2-IL-1β* transgenic mice have reduced expression of key regulators of immature myeloid cell inducers IL-17 and S100A8/a9 **A.** Relative IL-17A, IL-17C and S100A9 expression from esophageal and SCJ metaplasia tissues of *L2-IL-1β* and *K14-Cdx2::L2-IL-1β* mice. *n* = 3 mice in each group. Error bars display standard deviations; *p* values based on one-sided student *T*-testing comparing gene expression in *L2-IL-1β* and *K14-Cdx2::L2-IL-1β* mice; ‡ *p* = 0.164; *p = 0.049; **p = 0.036; ***p ≤ 0.018; *n* = 3 animals for each genotype, with each sample assayed in triplicate. **B.** Immunostaining for S100A9 in esophagi of *L2-IL-1β* and *K14-Cdx2::L2-IL-1β* mice. E; squamous epithelium; SM: submucosa. *n* = 3 mice in each group. **C.** Model for the onset of metaplasia in *L2-IL-1β* mice, and its inhibition in *K14-Cdx2::L2-IL-1β* mice. IMC: immature myeloid cells; Green arrows-enhancing activity, red lines-inhibitory function.

We next considered how the development and localization of CD11b^+^Gr-1^+^ cells were inhibited in the *K14-Cdx2::L2-IL-1β* mice. We re-examined the microarray data and identified cytokines IL-17a and 17c, and the immune modulatory S100A8 and S100A9 (Calgranulin-A and B) as being significantly diminished in the SCJ metaplasia (Table 1). All three factors have been demonstrated as key regulators promoting the development and release of immature myeloid CD11b^+^Gr-1^+^ lineage from the bone marrow [18, 21, 22]. Moreover, three of them, IL-17c, S100A8 and S100A9, are known to be expressed by squamous epithelium including the esophagus [23–27]. Quantitative PCR analysis of the expression of these genes in *L2-IL-1β* and *K14-Cdx2::L2-IL-1β* mice confirms a strong reduction of their mRNA levels in both the esophagus and SCJ nodular metaplasia in *K14-Cdx2::L2-IL-1β* mice (Figure 14A). IL-17a mRNA levels were most significantly reduced, by 6-fold (*p* = 0.013 and = 0.036, respectively; *n* = 3), while IL-17c levels were reduced by 60% to 50% of control levels (*p* = 0.036 and = 0.164; *n* = 3), and S100A9 mRNA levels were reduced by 5 and 2-fold, respectively (*p* = 0.018 and = 0.049; *n* = 3). In support of this, we performed immunohistochemistry for S100A9 protein in the esophagi from both *L2-IL-1β* and *K14-Cdx2::L2-IL-1β* transgenic mice. S100A9 protein is detected more abundantly in the squamous epithelium of *L2-IL-1β* when compared to *K14-Cdx2::L2-IL-1β* mice (Figure 14B). Additionally, S100A9 expressing cells were also much more abundant in the inflammatory cells infiltrating the submucosa of the *L2-IL-1β* mice than the *K14-Cdx2::L2-IL-1β* transgenic mice. Together with the qPCR data, we conclude that IL-17 and S100A8/A9 expression is diminished in the esophagi of *K14-Cdx2::L2-IL-1β* transgenic mice. Moreover, it suggests that the observed reduction in metaplasia development at the SCJ of *K14-Cdx2::L2-IL-1β* mice may be due to this diminished expression, as these proinflammatory signals are known to be required for the emergence of the CD8-suppressing CD11b^+^Gr-1^+^ immature myeloid cells [18, 21, 22].

## DISCUSSION

Our understanding of the mechanisms driving the development of Barrett's esophagus and its progression to esophageal adenocarcinoma has been limited by the paucity of animal models of these conditions. The development of the *L2-IL-1β* transgenic mouse model for intestinal metaplasia [7] has been a significant advance, and the studies we report here demonstrate the importance of animal models in advancing our understanding of disease mechanisms. Currently, the pathogenesis of Barrett's esophagus is thought to be a response to the chronic inflammation and injury from repeated gastric acid and bile acid reflux into the esophagus [4, 28]. The strength of the *L2-IL-1β* transgenic mouse as a model of BE and progression to EAC is that it is 1) dependent upon chronic esophageal inflammation, 2) that the metaplasia, which mimics BE at the level of morphology and gene expression, occurs at the SCJ as it does in BE, and 3) that bile acids can both accelerate disease progression and enhance intestinalization. Given this strength, our studies which elucidate a novel mechanism driving the onset of the metaplasia in these mice takes on even greater significance, providing new avenues of exploration for the human disease.

It is interesting to note that although conceptual models for the development of BE have for many years emphasized the role of reflux injury and inflammation in the development of the metaplasia, surprisingly few studies have deeply explored the inflammation and inflammatory cells associated with disease onset and progression. Proinflammatory cytokines including IFN-γ, IL-1β, IL-6, and IL-8, are expressed by epithelial cells in response to acid and bile reflux and drive the influx of a variety of proinflammatory cells including neutrophils, eosinophils, mast cells, macrophages as well as the adaptive immunity T and B cells [29, 30]. In the esophagus, activation of a T_H_1 pro-inflammatory response, characterized by production of interferon (IFN)-γ, is typical for acid reflux esophagitis [31]. Progression to BE is accompanied by a shift in cytokine expression patterns, including increased levels of interleukin IL-4, IL-5, IL-10, and IL-13, which are hallmarks of a T_H_2 humoral immune response [31–33]. Associated with this shift in cytokine patterns is a change in the infiltrating immune cells, with a reduction in macrophages in favor of T_H_2 associated plasma cells [34]. More recently, it was demonstrated that myeloid and plasmacytoid dendritic cells are recruited during the esophageal metaplasia-dysplasia-carcinoma sequence [35]. In the same report, it was demonstrated that cord-blood derived myeloid dendritic cells, when co-cultured with Barrett's esophagus and esophageal adenocarcinoma cell lines, displayed an immune tolerogenic behavior. This has direct bearing on our novel and important findings.

In humans, Cdx2 is expressed in BE and can be detected very early in the disease process, in the setting of reflux esophagitis [36, 37]. We anticipated that crossing our previously described *K14-Cdx2* mice with the *L2-IL-1β* transgenic mice might yield a more advanced, aggressive intestinal metaplasia. However, the opposite result was obtained. In our exploration of the mechanism for this protection, we eliminated trivial possibilities, such as Cdx2 expression reducing esophageal IL-1β levels and systemic inflammation, or that Cdx2 expression reduced cell proliferation, thereby limiting metaplasia formation. In contrast, we clearly demonstrate a very novel mechanism, that Cdx2 expression is associated with an increase in CD8^+^ cell-dependent apoptosis in the developing metaplasia. We demonstrate a very significant reduction in a subpopulation of immature myeloid cells with immune suppressor properties, known by the cell surface marking CD45^+^CD11b^+^Gr-1^+^. Based on the literature regarding these immature myeloid cells [12, 18], their loss or disruption *in vivo* typically leads to enhanced immune-mediated disruption of tumor growth, often via the actions of cytotoxic T-cells [38], consistent with our findings. We therefore speculate that with the loss of the immune-suppressing myeloid cells in the *K14-Cdx2::L2-IL-1β* mice, there is increased immune mediated surveillance and targeting of the abnormal metaplastic cells, leading to the increased apoptosis we observe in the metaplastic nodules at the SCJ. Evidence for the CD11b+Gr-1+ cells fulfilling this component of the model (Figure 7) is at present correlative but is fully consistent with the published literature on immature immune-suppressing myeloid cells. Formal testing of this mechanism is will be pursued in future experiments, including crossing the *L2-IL-1β* mice with IL-17 and S100A9 knock-out and S100A9 over-expressing mice.

An additional important question we do not fully answer here is the mechanism by which esophageal Cdx2 expression inhibits the development and recruitment of these immature myeloid cells. We clearly demonstrate that Cdx2 expression reduces levels of proinflammatory S100A8/A9 proteins and also IL-17 in the esophagus, but transgenic IL-1β is entirely unaffected. This is an important distinction, as S100A8/A9 and IL-17 are known from multiple studies to be critical for the induction of immature myeloid cells in cancer [12, 18]. Moreover, the S100A8/a9 and IL-17c proteins are expressed by squamous keratinocytes, esophageal epithelium, and Barrett's esophagus epithelium [23–27], and therefore may be directly affected by esophageal Cdx2. Together these findings suggest a Cdx2 expression in the esophagus reduces the production of the proinflammatory S100A8/a9 proteins and IL-17c (Figure 14).

However, elements of this mechanism remain unknown. Most critically is how the Cdx2 transgene yields this response, and this is a much harder question to address. We have explored *Cdx2's* role in intestinal biology [39–42] and observed that generally the transcription factor Cdx2 behaves as a transcriptional activator, not a repressor [10, 43]. We performed an *in silico* analysis of all three genes to determine if Cdx2 is known to bind their promoter regions using the ENCODE and TRANSFAC databases [44, 45] but found no evidence for an interaction with these genes by Cdx2. Moreover, we explored published genome-wide analyses of Cdx2 binding sites in intestinal epithelial cells and found no reports that Cdx2 protein bound and regulated IL-17, S100A8/A9 genes [46, 47].

It is interesting to note that there are several published reports exploring interactions between proinflammatory cytokines, NF-κB, and Cdx2. In gastric and cholangiocarcinoma cell lines, proinflammatory cytokines including IL-1β, IL-6, and TNF-α have all been reported to induce Cdx2 gene expression [48–50]. Several studies have even suggested this may be mediated by NF-κB binding to the Cdx2 promoter [51, 52]. In contrast, other studies have suggested Cdx2 may inhibit the NF-κB signaling pathway, possibly by binding the p65 subunit and inhibiting DNA binding of the NF-κB complex [53, 54]. We speculate that this latter mechanism may be how Cdx2 limits IL-17 and S100A8/9 expression. Until we clarify this mechanism, we cannot know whether Cdx2 plays a “protective” role, limiting disease progression to dysplasia and EAC by limiting the production of the immature myeloid cells. Therefore, this will remain an important focus for our future research efforts.

In many human cancers, immature myeloid cells have been under intense investigation due to their ability to promote tumor immune evasion and enhance tumorigenesis [12]. They are under investigation not only for the insights they yield into the pathogenesis of cancer, but for the potential therapeutic applications, including enhancement of anticancer immunotherapies [55]. Therefore, our work here, establishing their importance for disease onset in a mouse model for BE and EAC, is extremely significant not only for these new insights into BE pathogenesis our work provides, but also for potentially novel avenues of research and therapeutics which should now be now explored for patients with advanced BE and EAC.

## MATERIALS AND METHODS

### Animal studies

All studies with the mouse models were fully approved by the Institutional Animal Care and Use Committee (IACUC) at the University of Pennsylvania (IACUC#525400), and the animal care and use program conforms to all required standards. Mice were maintained in a specific pathogen-free facility with standard bedding and 12-hour light-dark cycles. The generation and genotyping of *K14-Cdx2* [6] and *L2-IL-1β* [7] transgenic mice have been previously described. *K14-Cdx2* transgenic mice were crossed with *L2-IL-1β* and yielded four genotypes analyzed for this study: *K14-Cdx2::L2-IL-1β* double transgenic, *L2-IL-1β* and *K14-Cdx2* single transgenic mice and corresponding wild type control as well. Mice were placed on drinking water containing deoxycholic acid (0.2% DCA, pH7.0) at age of 8 weeks. After 12-months of treatment, mice were sacrificed for analysis. The nodules were measured and excised for routine pathological examination. The following nodule indicators were evaluated in each mouse by iVision software: the number of mice with nodules as well as the numbers of and the calculated volumes of the nodules per mouse.

### Morphometric analysis

The squamocolumnar junction in HE-stained sections was evaluated for epithelial nodular hyperplasia, percentage of fields with mononuclear inflammatory infiltrates, neutrophilic inflammatory infiltrates, mucosal metaplasia, and mucosal lymphoid follicles. The extent of the histologic changes was assessed by determining the percentage of microscopic fields with positive criteria for lesions. All microscopic fields of each SCJ section were evaluated.

### Quantitative real-time PCR analysis

Samples were stored in tissue storage reagent (RNAlater; Ambion, Austin, TX). Total RNA was isolated using RNeasy Mini kit (Qiagen, Valencia, CA). cDNA was prepared from total RNA using the SuperScript^®^ VILO™ cDNA Synthesis Kit (Invitrogen, Carlsbad, CA). Primers were designed using Primer Express software (Applied Biosystems). Quantitative RT-PCR was performed on an ABI 7000 sequence detection system (Applied Biosystems, Foster City, CA), with SYBR green or Taqman as the fluorescent dye using standard PCR conditions. A dissociation curve was run with each PCR as a control. A ribosomal phosphoprotein, 36B4, was used as the normalization control.

The primer sequences used are as shown below: IL-1β forward 5′-CAAGCAACGACAAAATACCTGTG-3′; reverse 5′-AGACAAACCGTTTTTCCATCTTCT-3′;

IL-17A forward 5′-ATCCCTCAAAGCTCAGCGT GTC; reverse 5′-GGGTCTTCATTGCGGTGGAGAG-3′; IL-17C forward 5′-CCATGGAGATATCGCATCGA-3′; reverse 5′-GCATCCACGACACAAGCATT-3′; S100A9 forward 5′-CACAGTTGGCAACCTTTA TGA A-3′; reverse 5′-GGTCCTCCATGATGTCATTTATG-3′.

All the sequences of other primers used for real time PCR were described previously [6]. *p* values were determined by analysis of variance and Tukey rank mean test. ΔCt values were calculated after duplicate PCRs for each sample as described, and statistical analysis was performed. ΔΔCt values were then calculated and used to determine fold-change in expression.

### Immunohistochemical analysis

All specimens were isolated, rinsed in ice-cold PBS, fixed, and analyzed histologically by staining sections with hematoxylin and eosin (H&E) or immunohistochemically using standard methods as described [6]. Five-mm paraffin-embedded sections were pretreated with xylene and then placed in a microwave oven in 10 mmol/L citric acid buffer. Endogenous peroxidases were quenched using hydrogen peroxide before sections were incubated in avidin D blocking reagent and biotin blocking reagent. Primary antibodies used include rabbit monoclonal Cdx2 (1:500, Abcam), Caspase 3 (1:500, Cell Signaling), Rabbit polyclonal Muc2 (1:100, Santa Cruz), mouse monoclonal anti-CD45 (1:250, cell signaling), rat anti-Gr-1/NIMP-R14 (1:100, Abcam), mouse monoclonal MAC387 (1:1,000, Abcam), dendritic cell marker mouse monoclonal AP-MAB0801 (Abcam), rat anti-Ly-6G (1:1,000, BioXcell), Rabbit anti-Myeloperoxidase (1:500, Abcam), Rabbit anti- S100A9 (1:100, Novusbio). Sections were incubated with primary and biotinylated secondary antibodies and an avidin-horseradish peroxidase conjugate (Vectastain Elite ABC kit; Vector Laboratories, Burlingame, CA) following the manufacturer's protocol. The signal was developed using the 3,3-diaminobenzidine substrate kit (Vector Laboratories). Sections were counterstained with hematoxylin.

Alcian blue staining, slides were deparaffinized. After application of 3% aqueous acetic acid to the slides, 1% Alcian blue in 3% acetic acid, pH 2.5, was applied. Sections were washed and counterstained with 0.1% nuclear fast red, dehydrated, and mounted. For immunofluorescence detection, the tissue sections were incubated with primary antibody overnight at 4°C and secondary antibodies at 37°C for 30 minutes. After incubation, slides were washed with PBS three times, counterstained with DAPI, and then photographed with a Nikon E600 fluorescent microscope and confocal microscope.

### EdU cell proliferation assay

The transgenic and wild-type littermates were injected intraperitoneally with EdU (Life Technologies) 1 hour prior to sacrifice, esophagus and squamo-columnar junction area were harvested and embedded in paraffin. 5 μm thick sections were subjected to the Click-iT and subjected to the Click-iT EdU proliferation Assay (Life Technologies). EdU that had been incorporated into newly synthesized DNA was detected by Alexa Fluor 594 azide (red) and cell nuclei were stained with DAPI (blue) counterstain. All Images were captured at 10× and 20× magnification. Three random 10× fields were taken from each group of litter matched transgenic and wild type mice. The EdU positive proliferating cells were quantified and normalized to the total cell number in each field. The graph is generated from the average ratio (EdU/DAPI) of three 10× fields in each group.

### ELISA

The levels of mouse IL-6 in sera of the transgenic mice were determined using an ELISA kit (BD Company, San Diego, CA). Absorbance was measured at 450 nm by a Tecan plate reader, and the samples were analyzed by Magellan 7.1 SP software.

### TUNEL assay

Apoptosis in sections was performed using *In situ* cell death detection kit with TMR Red according to the manufacturer instructions (Roche, West Sussex, UK) (Roche #12 156 792 910) and stored at 4°C until analysis.

### RNA microarray analysis

Microarray analyses were performed on triplicate RNA samples of SCJ metaplasia nodules from mice to identify differentially expressed genes comparing *L2-IL-1β* forestomach and *K14-Cdx2::L2-IL-1β* forestomach. The total nodules from mouse SCJ were isolated and snap-frozen and stored at −80°C for RNA preparation. cDNA was transcribed using 5 μg total RNA (Affymetrix) and run on Affymetrix Mouse 1.0ST Affymetrix Arrays. The statistical test significance analysis of Microarrays was applied using a two-class unpaired analysis and differentially expressed genes were identified using a fold change cutoff of ≥1.5 (up or down). Gene expression differences were considered statistically significant if the *p*-value was less than 0.01. A global test was done as to whether the expression profiles differed between the classes by permuting the labels of which arrays corresponded to which classes. The false discovery rate was estimated to be less than 0.13%. Cluster analysis was performed with Cluster and Treeview software. Microarray files were submitted to the GEO repository; file GSE60320.

### Flow cytometry analysis

Single-cell suspensions of fresh esophagi, SCJ metaplasia nodules, or spleen) were prepared. Spleens were crushed and passed through a 70-μm cell strainer, treated with ACK Lysis Buffer (Invitrogen) and washed twice with RPMI/10% FCS. Esophagi and forestomachs were minced into 1–2 mm pieces, incubated in 1 mg/mL collagenase (Sigma-Aldrich) in RPMI for 45 minutes at 37°C, and then were passed through a 70-μm cell strainer and washed once RPMI/10% FCS. Cells were stained in PBS/0.5% FCS with the following antibodies: CD45 (Biolegend clone 30-F11), CD11c (clone HL3), anti-mouse CD45R (B220) FITC, CD3e (clone 145–2C11), CD49b (clone DX5), CD19 (clone 1D3) from eBioscience and Pacific Blue Ly-6G/Ly-6C (Gr-1) (clone RBG-8C5) from Biolegend, Anti-Mouse CD11b (clone M1/70) PE from eBiosciences, anti-Myeloperoxidase antibody [2D4] FITC from Abcam, F4/80 (clone C1:A3-1) from AbD Serotec. Flow cytometry was done using a BD FACSCanto or LSRII (BD Biosciences Immunocytometry Systems), and data were analyzed with BD FACSDiva software. Gates were set using negative controls and positive populations were corrected by subtraction of background and nonspecific binding of the antibody.

For CD8^+^ T-cell ablation studies, *K14-Cdx2::L2-IL1β* mice were injected on days one, three and five with 200 μg of anti-mouse CD8a (clone 53-6.72) or isotype control (clone 2A) antibodies from BioXcell. On the sixth day, blood, spleens and esophagi were collected for flow cytometry confirmation of decreased CD8^+^ cells. SCJ metaplasia sections were fixed for histology and TUNEL assays.

### *In vitro* T cells suppression assay

To evaluate the ability of CD11b+ Gr-1+ myeloid-derived suppressor cells (MDSC) isolated from the spleen from *L2- IL-1β* transgenic mice to suppress antigen specific T cell proliferation, we performed a T-cell proliferation assay. A single cell suspension of splenocytes isolated from wild-type mice was made by homogenizing spleen with a 1 ml syringe through a 70 μM filter into a 50 ml conical tube. Red blood cells were lysed using lysis solution and quenched with HBSS. Red blood cell depleted murine splenocytes were resuspended at 1 × 10^6^/ml and labeled with 5 μM green fluorochrome carboxyfluorescein succinimidyl ester (CFSE). Cells were activated with anti-CD3/CD28 coated beads (Gibco, Life technologies) and seeded in triplicate and cultured in RPMI-1640 supplemented with 10% fetal bovine serum. Splenocytes were cultured either alone or in the presence of CD11b^+^Gr-1^+^ cells isolated from *L2-IL-1β* mice at different ratios 0:1, 1:1, 1:2, 1:4, 1:8, 1:16. After 72 h, cells were collected and stained with 7AAD, anti-CD45, CD3, CD4, Gr-1 and CD11b mAbs cocktail. Proliferation was determined by CFSE dilution and flow cytometric analysis on a FACSLSRII cytometer (BD Biosciences) with initial gating on the CD3^+^/CD4^+^ populations.

### Statistical analysis

GraphPad Prism version 3.04 was used for all statistical analyses (GraphPad, San Diego, CA, USA). For power analysis GraphPad Statmate or G*power3 was used. All data are represented as mean and standard deviation unless otherwise stated with *p*-value cutoff of ≤ 0.05 unless otherwise indicated. Replicate numbers and specific statistical tests employed are indicated in the figure legends.

All authors had access to the study data and had reviewed and approved the final manuscript.

## SUPPLEMENTARY TABLE


