# Supplementary material for: Immature myeloid progenitors promote disease progression in a mouse model of Barrett's-like metaplasia
Source: Oncotarget. 2015 Oct 12;6(32):32980–3005. doi: 10.18632/oncotarget.5431 (PMC4741744; doi:10.18632/oncotarget.5431)
Supplement: Supplementary file 1 [file oncotarget-06-32980-s001.pdf]

SUPPLEMENTARY TABLE

Supplementary Table S1: Microarray results containing 199 genes whose expression differed by 2-fold or more and had less than a 10% false discovery rate

Gene Symbol	RefSeq	Fold-Change (Cdx2/IL-1B vs. IL-1B)	*q*-value (%)	Score (d)	*p*-value (Cdx2/IL-1B vs. IL-1B)
Uox	ENSMUST00000029837	−14.9333	0	−15.0767	6.51E-05
Akr1c18	NM_134066	−10.1761	1.47185	−4.86307	0.00691962
A630038E17Rik	BC111819	−7.20884	0	−16.1486	3.72E-05
A630038E17Rik	BC111819	−7.03042	0	–9.93134	0.000353194
Gm5416	NM_001082542	−6.21167	1.29192	−4.94736	0.00616605
Vnn3	NM_011979	−5.61856	0	–9.77786	0.000353637
Muc5b	NM_028801	−5.61801	1.87105	−4.11052	0.0121366
Tmprss11bnl	ENSMUST00000038448	−5.30621	0	−15.1088	4.38E-05
Lpo	NM_080420	−5.22939	0	−7.51084	0.00109903
Ifi202b	NM_008327	−5.07291	3.61419	−3.34152	0.0246727
Pyhin1	NM_175026	−4.92285	1.47185	−4.87958	0.00627679
Atp12a	NM_138652	−4.90446	2.40305	−4.0791	0.0122654
	---	−4.69872	1.87105	−4.09897	0.0119785
Sprr2g	NR_003548	−4.69418	4.68177	−3.10933	0.0310129
Tmprss11g	NM_177162	−4.28837	0	−15.2387	3.58E-05
Atp13a4	NM_001164612	−4.25089	1.05914	−5.86038	0.00293742
Tcrg-V6	Z48592	−4.23609	1.05914	−5.87465	0.00290585
Sptlc3	NM_175467	−4.22396	0	−8.16011	0.000713163
	---	−4.09536	1.05914	−5.50774	0.00374389
Slc5a8	NM_145423	−3.99601	0	−6.94518	0.00141248
Stfa3	NM_025288	−3.98957	0	−7.56497	0.000973656
Olfm4	NM_001030294	−3.96623	0	−6.69396	0.00164929
Wfdc12	NM_138684	−3.96555	2.80504	−3.91421	0.0137842
Defb3	NM_013756	−3.94574	5.35637	−2.99139	0.0344597
Tnfrsf9	NM_011612	−3.75936	0	–8.064	0.000714102
S100a9	ENSMUST00000117167	−3.73631	0	−11.5274	0.000132201
Cyp7b1	NM_007825	−3.68619	0	−13.3532	6.20E-05
Pdcd1	NM_008798	−3.55437	0	−10.2341	0.000227893
Sprr2 h	NM_011474	−3.51013	5.35637	−2.9931	0.0338806
BC100530	ENSMUST00000096089	−3.42958	1.05914	–5.62096	0.00326355
	---	−3.33596	1.47185	−4.56878	0.00742709

(*Continued*)

Gene Symbol	RefSeq	Fold-Change (Cdx2/IL-1B vs. IL-1B)	*q*-value (%)	Score (d)	*p*-value (Cdx2/IL-1B vs. IL-1B)
S100a8	NM_013650	−3.28772	0	–7.66234	0.000837659
1190003J15Rik	ENSMUST00000026554	−3.28515	1.47185	−4.70993	0.00656509
Ear1	NM_007894	−3.26191	1.47185	−4.69779	0.00661719
Ifitm1	ENSMUST00000106042	−3.21242	0	–7.63403	0.000840821
	---	−3.20719	1.21514	–5.40404	0.00374508
	---	−3.16964	1.47185	−4.61284	0.00704579
Mug1	NM_008645	−3.16554	5.98878	−2.76435	0.0428922
Pglyrp4	NM_001165968	−3.14416	1.05914	−6.1311	0.00218518
Cxcr6	NM_030712	−3.08408	1.21514	−5.15375	0.00447996
	---	−3.05062	0	−7.40276	0.000936712
Sprr1b	NM_009265	−3.04819	2.40305	−3.98401	0.0121922
Il1b	NM_008361	−3.04682	1.87105	−4.22259	0.0097923
	---	−3.02315	1.05914	−6.10626	0.00218217
2010107G12Rik	NM_001025573	−2.91426	0	−6.8816	0.00126484
	---	−2.88069	1.47185	−4.78103	0.00590767
Il17a	NM_010552	−2.87594	1.05914	–5.69108	0.00287537
Slc1a1	ENSMUST00000025875	−2.85519	1.47185	−4.63416	0.0066685
Slc6a14	NM_020049	−2.85356	1.05914	–6.34742	0.00178622
Gm1045	NM_001177577	−2.82565	0	−10.1915	0.000189406
Csf3r	NR_045561	−2.8235	3.28523	−3.52858	0.0185792
Sprr2f	NM_011472	−2.82333	1.70914	−4.25596	0.00926724
Il17c	NM_145834	−2.77006	6.73037	−2.60104	0.050553
Saa3	NM_011315	−2.76797	2.96978	−3.61786	0.0168901
Cd3g	NM_009850	−2.76493	8.92894	−2.34912	0.0680025
Lcn2	NM_008491	−2.76479	0	−6.69865	0.00137978
Tmprss11e	NM_172880	−2.74916	5.98878	−2.8152	0.039523
Tmigd1	NM_025655	−2.74252	1.21514	–5.23439	0.00399142
Slc37a2	NM_001145960	−2.73427	8.92894	−2.44295	0.0607078
Defb14	NM_183026	−2.72681	4.68177	−3.1555	0.0271445
	---	−2.72273	2.82036	−3.81971	0.0137776
Chi3l1	NM_007695	−2.71049	6.73037	−2.64554	0.0478296
Gm15987	NR_045009	−2.70602	4.00359	−3.27134	0.0239514
Sprr2k	NM_011477	−2.69383	1.29192	–5.00621	0.00475751
Snora75	NR_028478	−2.68927	2.96978	−3.67346	0.0158413
	---	−2.68323	1.63853	−4.41833	0.00785399

(*Continued*)

Gene Symbol	RefSeq	Fold-Change (Cdx2/IL-1B vs. IL-1B)	*q*-value (%)	Score (d)	*p*-value (Cdx2/IL-1B vs. IL-1B)
Clec4e	NM_019948	−2.67522	1.05914	–6.06005	0.00210464
Cdhr1	NM_130878	−2.62858	3.28523	−3.51707	0.0184085
Mmp9	NM_013599	−2.62158	4.00359	−3.31275	0.0227285
Ces1 h	AK009689	−2.62027	2.96978	−3.66076	0.0159068
Nox1	ENSMUST00000033610	−2.61431	1.47185	−4.66675	0.00624271
Klk12	ENSMUST00000107970	−2.59694	1.21514	–5.20563	0.00396831
Mmp3	NM_010809	−2.58433	5.98878	−2.75288	0.0418639
Ighv1–47	X14623	−2.57879	1.05914	−5.59985	0.00289786
Gpr110	ENSMUST00000047399	−2.54291	2.40305	−4.03839	0.0108918
Klri2	NM_177155	−2.52273	0	−10.826	0.000118517
Ugt8a	NM_011674	−2.50342	5.35637	−2.91846	0.0344439
	---	−2.49891	1.87105	−4.15996	0.00964081
Ptk6	ENSMUST00000016511	−2.49235	1.70914	−4.27699	0.00863885
Slc16a14	ENSMUST00000027422	−2.48668	9.80618	−2.246	0.0757406
Tcrg-V5	Z48591	−2.47034	1.70914	−4.28833	0.00851363
Gpr65	NM_008152	−2.46627	1.47185	−4.65837	0.00610487
Klrc1	ENSMUST00000032271	−2.46607	0	−8.43156	0.000419897
Lgi2	NM_144945	−2.46469	0	−7.14023	0.000937801
Defa-ps12	NR_002878	−2.45884	3.28523	−3.48467	0.0186069
Adcy8	NM_009623	−2.44958	1.47185	−4.80111	0.00537414
Cd3e	NM_007648	−2.44474	2.96978	−3.57071	0.0169952
Prss22	NM_133731	−2.43784	2.82036	−3.8101	0.0133476
Tmprss11d	ENSMUST00000031175	−2.43425	0	−8.04725	0.000520412
Havcr2	NM_134250	−2.42003	1.05914	−6.51103	0.00141451
Alpl	NM_007431	−2.36924	0	−10.9783	9.83E-05
Ces1g	ENSMUST00000044602	−2.3609	6.73037	−2.68032	0.0445328
Krt6a	NM_008476	−2.34649	1.29192	−4.91403	0.00475121
Defb6	NM_054074	−2.33926	9.80618	−2.31371	0.0689038
Il2rb	NM_008368	−2.33622	0	−13.1679	3.26E-05
Klk14	NM_174866	−2.33083	1.05914	–5.442	0.0030575
Cldn8	NM_018778	−2.31595	9.80618	−2.26279	0.0731959
Snora81	NR_034048	−2.31443	1.47185	−4.56728	0.00637787
Ankk1	NM_172922	−2.30711	1.29192	−5.01905	0.00429619
Moxd1	NM_021509	−2.30548	2.80504	−3.87954	0.0121489
Blk	NM_007549	−2.29922	1.47185	−4.75843	0.00536239
Cst7	NM_009977	−2.28314	1.70914	−4.30757	0.00801877

(*Continued*)

Gene Symbol	RefSeq	Fold-Change (Cdx2/IL-1B vs. IL-1B)	*q*-value (%)	Score (d)	*p*-value (Cdx2/IL-1B vs. IL-1B)
Ambp	NM_007443	−2.26612	8.92894	−2.34231	0.0660631
Expi	NM_007969	−2.25922	0	−9.86684	0.000162159
Fam167a	NM_177628	−2.25793	0	−7.85868	0.000532401
Nat8l	NM_001001985	−2.25621	1.21514	−5.12827	0.00385432
Snora15	NR_003681	−2.25342	5.35637	−2.84585	0.0362549
Stap1	ENSMUST00000031171	−2.24255	2.80504	−3.87324	0.0120524
Spink12	ENSMUST00000081271	−2.23276	4.00359	−3.23267	0.0234261
Aipl1	NM_053245	−2.22704	1.29192	−4.97417	0.00434818
A630038E17Rik	BC038285	−2.22224	2.82036	−3.72491	0.0139225
Dpep2	ENSMUST00000150001	−2.21706	2.82036	−3.78871	0.0130361
Ptpn22	NM_008979	−2.19618	1.21514	–5.39783	0.00301512
Esr1	NM_007956	−2.18994	4.68177	−3.12162	0.0262641
Tdh	NM_021480	−2.18358	1.70914	−4.35323	0.00747082
Il27ra	NM_016671	−2.18232	0	−7.54608	0.000621474
2300002M23Rik	ENSMUST00000044326	−2.17384	2.82036	−3.7647	0.0132144
	---	−2.17087	8.15245	−2.47817	0.055352
Selplg	NM_009151	−2.1509	2.40305	−3.97338	0.010656
Gzmb	NM_013542	−2.14237	1.47185	−4.83056	0.00477608
Il23a	NM_031252	−2.13829	1.05914	–6.30409	0.00144615
Pion	NM_175437	−2.13423	1.87105	−4.16832	0.00876621
Pglyrp1	NM_009402	−2.12096	2.82036	−3.71678	0.0136792
Bhlhe22	NM_021560	−2.11237	0	−7.42563	0.000641588
Mab21l3	ENSMUST00000118411	−2.1097	1.47185	−4.7535	0.00504888
	---	−2.10837	2.40305	−4.0647	0.00961486
Tnfaip2	ENSMUST00000102745	−2.104	2.40305	−4.04434	0.0097947
Trem1	NM_021406	−2.09072	0	–6.75928	0.00100605
P2ry2	NM_008773	−2.08968	1.47185	−4.88689	0.00444745
Trim30b	NM_175648	−2.0863	1.63853	−4.5019	0.00628989
Shisa3	NM_001033415	−2.07089	2.82036	−3.73879	0.0131839
Upp1	NM_001159402	−2.05616	1.63853	−4.4824	0.00632832
Kcnh1	NM_010600	−2.0543	1.70914	−4.40111	0.00682478
	---	−2.05376	0	–9.86367	0.000131604
Krt16	NM_008470	−2.05349	1.29192	−5.03807	0.00383199
Gm11992	NM_001037928	−2.0525	6.73037	−2.69238	0.0419691
Cd53	NM_007651	−2.04865	1.21514	−5.3718	0.00287589
Eya1	NM_010164	−2.03747	2.80504	−3.8923	0.011149

(*Continued*)

Gene Symbol	RefSeq	Fold-Change (Cdx2/IL-1B vs. IL-1B)	*q*-value (%)	Score (d)	*p*-value (Cdx2/IL-1B vs. IL-1B)
Abpb	NM_001100464	−2.03699	1.47185	−4.60805	0.00558745
Dsc2	ENSMUST00000039247	−2.03529	0	–8.68074	0.000264253
F2rl2	ENSMUST00000022182	−2.0338	0	–8.03007	0.000401129
H2-Q10	ENSMUST00000040279	−2.03213	9.80618	−2.27067	0.0701467
Gm20317	XR_105322	−2.03175	1.70914	−4.27253	0.00764419
	---	−2.02793	5.35637	−2.95088	0.0308161
Tgm5	ENSMUST00000028721	−2.02783	2.80504	−3.95365	0.0104466
Scarna3b	NR_028544	−2.01915	2.82036	−3.80108	0.0121583
Icos	NM_017480	−2.01872	3.28523	−3.53098	0.0161314
Steap4	NM_054098	−2.01622	5.98878	−2.8247	0.0356098
	---	−2.01291	3.61419	−3.39604	0.0186242
	---	−2.01291	3.61419	−3.39604	0.0186242
	---	−2.0116	8.92894	−2.35365	0.0630274
	---	−2.00012	1.70914	−4.25051	0.00770704
Ada	ENSMUST00000017841	2.01162	2.80504	4.86559	0.00437207
Bves	NM_024285	2.03721	8.15245	3.1188	0.0254933
Rap1gap2	ENSMUST00000102521	2.03779	8.15245	3.1837	0.0237029
Lce1b	NM_026822	2.03913	8.15245	3.18367	0.023711
	---	2.03999	1.29192	6.89905	0.000877053
Slc38a3	NM_023805	2.04733	5.98878	3.39222	0.0188875
Fmo2	ENSMUST00000045902	2.06078	6.73037	3.23332	0.0225604
Lce1f	NM_026394	2.08208	9.80618	2.81662	0.0364474
Aldh3a1	NM_007436	2.09045	8.15245	3.08554	0.0267946
Ftl1	NM_010240	2.0905	8.92894	2.96144	0.0308625
Lce6a	NM_001166172	2.11126	5.27599	3.53637	0.0164686
Lce1l	NM_028628	2.11625	4.68177	3.8239	0.0122509
Hmgcs2	NM_008256	2.16739	1.29192	7.48554	0.000640253
Slc25a23	NM_025877	2.17316	2.96978	4.36867	0.00733975
Slc2a4	NM_009204	2.19669	8.15245	3.15847	0.0252449
Hsd17b14	NM_025330	2.20029	5.27599	3.77199	0.0132041
	---	2.21057	9.80618	2.80521	0.0377557
Cpa6	NM_177834	2.21236	4.68177	3.86008	0.0121225
Gpr37	ENSMUST00000054867	2.25629	1.43956	6.18284	0.00167856
Mboat2	NM_026037	2.30766	8.15245	3.1756	0.0252514
Pcp4l1	NM_025557	2.31921	5.98878	3.37713	0.0203672
Pcp4	NM_008791	2.32222	5.27599	3.5736	0.0165909

(*Continued*)

Gene Symbol	RefSeq	Fold-Change (Cdx2/IL-1B vs. IL-1B)	*q*-value (%)	Score (d)	*p*-value (Cdx2/IL-1B vs. IL-1B)
St3gal5	NM_011375	2.33146	5.98878	3.37878	0.0203747
Gldc	NM_138595	2.34998	1.87105	5.46355	0.00302381
Inmt	ENSMUST00000003569	2.35124	5.98878	3.3848	0.0203125
Spp1	NM_001204201	2.4025	6.73037	3.35821	0.0210589
Gm13124	NM_001085542	2.43798	3.61419	4.17312	0.00941418
5430427M07Rik	NR_045858	2.48309	5.98878	3.45904	0.0191744
2310050C09Rik	NM_025621	2.49507	4.68177	3.83691	0.0131255
Adcyap1r1	NM_007407	2.56201	5.27599	3.66685	0.0156873
	---	2.58072	8.92894	3.04785	0.0300768
Snord13	NR_028522	2.59187	8.15245	3.2049	0.0254013
	---	2.66855	8.92894	2.98721	0.0324214
Tacr2	NM_009314	2.67941	2.96978	4.57304	0.00684823
E330013P06	NR_045402	2.74475	6.73037	3.28414	0.0237194
Trim50	ENSMUST00000065785	2.75183	9.80618	2.86964	0.0371922
Esrrg	ENSMUST00000110938	2.80439	8.92894	2.99189	0.0326175
Lce1m	NM_025420	2.98417	1.29192	7.31878	0.000972791
Krt79	NM_146063	3.71593	3.61419	4.16121	0.0108772
Lce1k	NM_001254760	3.73066	8.15245	3.19856	0.0275127
5430419D17Rik	NM_175166	3.77612	1.05914	9.38113	0.000358072
Mir145	NR_029557	3.99754	3.61419	4.10849	0.0115672
Far2	NM_178797	5.33113	1.43956	5.8836	0.00304483
Ifi27l2b	NM_145449	6.07215	2.96978	4.5413	0.00849458
Cdx2	ENSMUST00000031650	6.622	1.05914	9.52289	0.000418314
Cdhr5	NM_001114322	7.18268	3.61419	4.18042	0.0117104
Sycn	NM_026716	13.5724	2.40305	4.99092	0.00640984
